# Comparative Effectiveness of Cholesteryl Ester Transfer Protein (CETP) Inhibitors on Lipid Profiles in Adults With Hyperlipidemia: A Comprehensive Systematic Review and Frequentist Network Meta‐Analysis of Randomized Controlled Trials

**DOI:** 10.1002/clc.70204

**Published:** 2025-09-14

**Authors:** Ibrahim Khalil, M. Rafiqul Islam, Sunjida Amin Promi, Arindam Das Joy, Md Abu Sayed, Durjoy Acharjee, Ali Saad Al‐shammari, Sakib Abrar, Ta‐Seen Bin Jamil, Malaika Taseen, Suborna Biswas, Sumaya Khan Mifty, Sajjad Ghanim Al‐Badri, Avijit Debnath, Md. Imran Hossain, Mahmuda Akter

**Affiliations:** ^1^ Dhaka Medical College and Hospital Dhaka Bangladesh; ^2^ Shaheed Suhrawardy Medical College and Hospital Dhaka Bangladesh; ^3^ Chattogram Medical College Chattogram Bangladesh; ^4^ College of Medicine University of Baghdad Baghdad Iraq; ^5^ Gazi Medical College Khulna Bangladesh; ^6^ Jalalabad Ragib‐Rabeya Medical College and Hospital Sylhet Bangladesh; ^7^ Manikganj Medical College Manikganj Bangladesh

**Keywords:** anacetrapib, CETP inhibitors, hyperlipidemia, network meta‐analysis, obicetrapib, statins

## Abstract

**Background:**

Hyperlipidemia, a key risk factor for cardiovascular disease, is characterized by elevated low‐density lipoprotein cholesterol (LDL‐C), triglycerides, and reduced high‐density lipoprotein cholesterol (HDL‐C). Cholesteryl ester transfer protein (CETP) inhibitors, such as anacetrapib, obicetrapib, evacetrapib, dalcetrapib, and torcetrapib, aim to improve lipid profiles by increasing HDL‐C and reducing LDL‐C, but their comparative efficacy remains unclear.

**Methods:**

This systematic review and frequentist network meta‐analysis, conducted per PRISMA‐NMA guidelines, included 33 randomized controlled trials (RCTs) involving 120,292 adults with hyperlipidemia. We compared CETP inhibitors, alone or with statins, against placebo or other lipid‐lowering therapies. Primary outcome was LDL‐C reduction; secondary outcomes included HDL‐C, triglycerides, and total cholesterol changes. Random‐effects models calculated mean differences (MD) with 95% confidence intervals (CI), and P‐scores ranked interventions.

**Results:**

Atorvastatin + obicetrapib showed the largest reduction in LDL‐C levels (MD: −69.00, 95% CI: −95.96 to −42.04, *p* < 0.0001), followed by rosuvastatin + obicetrapib (MD: −60.70, 95% CI: −99.28 to −22.12, *p* = 0.0020). Atorvastatin + obicetrapib yielded highly significant increase in HDL‐C levels (MD: 149.90, 95% CI: 121.70 to 178.10, *p* < 0.0001), but rosuvastatin + obicetrapib showed the greatest increase (MD: 158.90, 95% CI: 118.59 to 199.21, *p* < 0.0001) and obicetrapib monotherapy (MD: 139.00, 95% CI: 129.05 to 148.96, *p* < 0.0001), while rosuvastatin + evacetrapib led triglyceride reductions (MD: −31.70 mg/dL). Rosuvastatin was most effective for total cholesterol (MD: −31.60 mg/dL).

**Conclusion:**

CETP inhibitors, particularly anacetrapib and obicetrapib combined with statins, significantly improve lipid profiles, offering potential therapeutic benefits for hyperlipidemia management and cardiovascular risk reduction.

**Trial Registration:** The study was registered with **PROSPERO** to ensure transparency and adherence to methodological rigor (Registration ID: CRD420250652666).

## Summary

- Network meta‐analysis compares CETP inhibitors’ effects on lipid profile.
- Atorvastatin + obicetrapib tops LDL‐C reduction in hyperlipidemia.
- Rosuvastatin + obicetrapib markedly boosts HDL‐C levels.
- Rosuvastatin + evacetrapib leads in triglyceride reduction.
- Rosuvastatin excels in lowering total cholesterol.

## 1 Introduction

Hyperlipidemia is a major modifiable risk factor for cardiovascular disease (CVD), characterized by abnormal lipid levels, including elevated low‐density lipoprotein cholesterol (LDL‐C) and triglycerides, alongside reduced high‐density lipoprotein cholesterol (HDL‐C) [1]. These lipid imbalances contribute to atherosclerosis, the underlying cause of many cardiovascular events. Current therapies primarily target LDL‐C and triglycerides; however, pharmacologically increasing HDL‐C has proven challenging due to limited efficacy and safety concerns [2].

Cholesteryl ester transfer protein (CETP) plays a pivotal role in lipid metabolism by transferring cholesteryl esters from HDL to LDL and very low‐density lipoprotein (VLDL) particles in exchange for triglycerides. This process not only lowers HDL‐C levels but also raises LDL‐C and VLDL cholesterol, thereby promoting atherogenesis [2, 3]. CETP activity also affects apolipoprotein levels, decreasing apolipoprotein A‐I (ApoA‐I), the key protein in HDL responsible for reverse cholesterol transport, while increasing apolipoprotein B (ApoB), the structural protein of atherogenic particles such as LDL and VLDL. Elevated ApoB levels strongly predict cardiovascular risk, reflecting the burden of atherogenic lipoproteins [3].

CETP inhibitors, including torcetrapib, anacetrapib, evacetrapib, and dalcetrapib, were developed to block CETP activity, resulting in increased HDL‐C and reductions in LDL‐C, total cholesterol, and ApoB levels. However, the early promise of torcetrapib was overshadowed by off‐target effects, including elevated blood pressure, electrolyte imbalances, and increased cardiovascular mortality, leading to its discontinuation [4]. These setbacks underscored the need for next‐generation CETP inhibitors with improved safety profiles, such as anacetrapib and evacetrapib, which have demonstrated more favorable lipid‐modifying effects but have yielded inconsistent reductions in cardiovascular events. Dalcetrapib has shown limited efficacy, further highlighting the mixed outcomes within this drug class [5].

Despite these challenges, CETP inhibitors remain of interest due to their unique mechanism of action and potential to address residual cardiovascular risk. Their effects on HDL‐C, LDL‐C, triglycerides, and apolipoproteins suggest they may offer additional benefits, particularly when used in combination with statins. However, their role in optimizing cardiovascular outcomes remains unclear [6, 7, 8]. To comprehensively evaluate the efficacy of CETP inhibitors, this systematic review employs a frequentist network meta‐analysis, a robust statistical approach that allows for the comparison of multiple interventions across diverse trials. This methodology will assess not only the effects of CETP inhibitors on lipid parameters (LDL‐C, HDL‐C, triglycerides, and total cholesterol) but also their potential benefits when used as monotherapy or in combination with statins.

## 2 Methods

### 2.1 Study Design

This network meta‐analysis was conducted in accordance with the Preferred Reporting Items for Systematic Reviews and Meta‐Analyses extension for network meta‐analysis (PRISMA‐NMA) guidelines [9, 10, 11], and the Cochrane Handbook for Systematic Reviews of Interventions [12, 13]. The primary aim of the study was to evaluate and compare the effectiveness of Cholesteryl Ester Transfer Protein (CETP) inhibitors in managing lipid profiles in adults, with a focus on key lipid parameters such as LDL‐C, HDL‐C, total cholesterol, and triglycerides. The study was registered with PROSPERO (Registration ID: CRD420250652666) to ensure methodological rigor and transparency [14].

### 2.2 Eligibility Criteria

The inclusion criteria for studies were carefully defined. Only randomized controlled trials (RCTs) comparing CETP inhibitors to placebo or other interventions in adults were considered. The study population included adults aged 18 years and older who were diagnosed with hyperlipidemia, including those with primary hyperlipidemia, hypercholesterolemia, or hypertriglyceridemia. The intervention of interest was the use of CETP inhibitors, such as anacetrapib, evacetrapib, obicetrapib, and dalcetrapib, either as monotherapy or in combination with statins or other lipid‐lowering therapies. The comparator group consisted of placebo, standard care, or other lipid‐lowering agents not targeting CETP. The outcomes required for inclusion were the primary outcome of LDL‐C reduction and secondary outcomes such as total cholesterol, HDL‐C, and triglycerides. Only peer‐reviewed journal articles published in English were included, and studies published up to January 1, 2025, were considered.

The exclusion criteria were also clearly defined to filter out irrelevant studies. Non‐randomized studies, observational studies, case reports, case series, reviews, meta‐analyses, editorials, and letters were excluded. Studies that did not report relevant lipid measures or clinical outcomes, non‐English publications, unpublished studies, conference abstracts, grey literature, and studies with overlapping patient populations were also excluded.

### 2.3 Information Sources

A comprehensive search was conducted across multiple electronic databases, including PubMed/Medline, Embase, Scopus, Cochrane Central Register of Controlled Trials, and ClinicalTrials. gov. Additional studies were identified through reference lists of relevant articles and ongoing clinical trials.

### 2.4 Search Strategy

The search strategy was structured around four primary concepts: CETP inhibitors, lipid profiles, hyperlipidemia, and randomized controlled trials (RCTs). The search terms used were designed to capture all relevant studies on the effects of CETP inhibitors on lipid profiles in patients with hyperlipidemia. For CETP inhibitors, MeSH terms such as “Cholesteryl Ester Transfer Protein Inhibitors”[Mesh] and keywords like “CETP Inhibitors”[Title/Abstract], “Anacetrapib”[Title/Abstract], “Obicetrapib”[Title/Abstract], “Torcetrapib”[Title/Abstract], “Evacetrapib”[Title/Abstract], and “Dalcetrapib”[Title/Abstract] were used. For lipid profiles, MeSH terms such as “Lipids”[Mesh], “Cholesterol”[Mesh], “Triglycerides”[Mesh] and keywords like “LDL‐C”[Title/Abstract], “HDL‐C”[Title/Abstract], “Lipid Profile”[Title/Abstract] were employed. Hyperlipidemia was captured using the MeSH terms “Hyperlipidemias”[Mesh], “Hypercholesterolemia”[Mesh], and keywords like “Hyperlipidemia”[Title/Abstract], “Dyslipidemia”[Title/Abstract]. For randomized controlled trials, the MeSH term “Randomized Controlled Trial”[Publication Type] and keywords like “RCT”[Title/Abstract], “Randomized Controlled Trials”[Mesh] were used. Boolean operators were applied to combine these concepts. Only studies involving human participants and published in English were included. Additional searches for clinical trials and conference abstracts were also conducted.

### 2.5 Study Selection

The study selection process was carried out in multiple stages to ensure the inclusion of high‐quality studies. Initially, two independent reviewers screened the titles and abstracts of all identified studies to assess their relevance. Full‐text articles of studies that passed the initial screening were retrieved and evaluated for eligibility by the same reviewers. Any discrepancies in study selection were resolved through discussion or consultation with a third reviewer.

### 2.6 Data Extraction

Data extraction was performed independently by two blinded reviewers using a standardized form. The data extracted from each study included study characteristics such as author, year, country, and study design. Participant characteristics such as sample size, age, gender distribution, and the type of hyperlipidemia were recorded. Intervention details, including the type, dosage, and administration of CETP inhibitors, were extracted. Comparator details, such as the description of placebo or control treatments, were also recorded. Finally, the primary outcome (LDL‐C reduction) and secondary outcomes (total cholesterol, HDL‐C, triglycerides) were documented.

### 2.7 Data Synthesis and Statistical Analysis

The primary outcome of interest was the percentage mean change in LDL‐C levels. Mean differences (MD) in percentage change from baseline, with 95% confidence intervals (CIs), were calculated using a random‐effects model. Secondary outcomes, including mean differences for total cholesterol, HDL‐C, and triglycerides, were also calculated using random‐effects models. A random‐effects model was chosen to account for potential study heterogeneity. Heterogeneity was assessed using Cochran's Q test and the I² statistic [15]. Additionally, P‐scores were calculated to rank the relative effectiveness of treatments, with higher P‐scores indicating greater effectiveness. Sensitivity analyses were performed to assess the robustness of the findings. Statistical analyses were conducted using R (version 4.4.3) with the netmeta, meta, and metafor packages for network meta‐analysis. Visualization of results was performed using the ggplot2 package, and Python (version 3.13) was used for additional data processing, visualization, and analysis.

### 2.8 Risk of Bias Assessment

The risk of bias in the included RCTs was assessed using the Cochrane Risk of Bias Tool 2. The key domains assessed included random sequence generation, allocation concealment, blinding, incomplete outcome data, and selective reporting [16, 17].

### 2.9 Assessment of Publication Bias

Publication bias was visually assessed using funnel plots. If asymmetry was detected, Egger's test was used to quantitatively evaluate the potential for bias for outcomes containing more than 10 studies. If significant publication bias was identified, additional sensitivity analyses were performed to confirm the findings.

## 3 Results

### 3.1 Characteristics of Included Studies

This network meta‐analysis included 33 randomized controlled trials (RCTs) with 120,292 participants evaluating cholesteryl ester transfer protein (CETP) inhibitors (anacetrapib, evacetrapib, dalcetrapib, obicetrapib, torcetrapib) versus placebo, statins, or other lipid‐lowering therapies in adults with hyperlipidemia. Trial sizes ranged from 10 to 15,225 participants per arm. The population, primarily middle‐aged to older adults (mean age 45.2–67.8 years, SD 9.46–12.9), was predominantly male (29.3–95%, median 77%), reflecting cardiovascular disease epidemiology. Baseline lipid profiles showed significant variability: LDL‐C ranged from 61 to 162.3 mg/dL (SD 15–23.3), HDL‐C from 32 to 58.8 mg/dL (SD 7–11.9), total cholesterol from 142.4 to 238.5 mg/dL (SD 19.7–31.5), and triglycerides from 42.54 to 205 mg/dL (SD 23.20–88), indicating diverse hyperlipidemia severity. Interventions included CETP inhibitors as monotherapy or combined with statins (atorvastatin 10–80 mg, rosuvastatin 10 mg, simvastatin 40 mg) or ezetimibe. CETP inhibitor doses varied: anacetrapib (10–300 mg), evacetrapib (30–500 mg), dalcetrapib (600–900 mg), obicetrapib (1–10 mg), and torcetrapib (10–120 mg). Comparators included placebo or statin‐based regimens. This diverse cohort enables robust evaluation of CETP inhibitors’ efficacy across lipid parameters in hyperlipidemia management.

**Figure 1:**
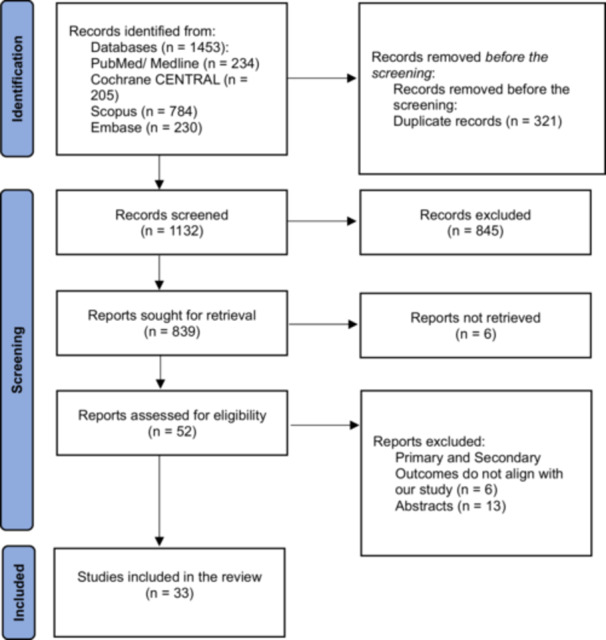
PRISMA 2020 flow diagram for systematic reviews, which included searches of databases and registers.

**Figure 2:**
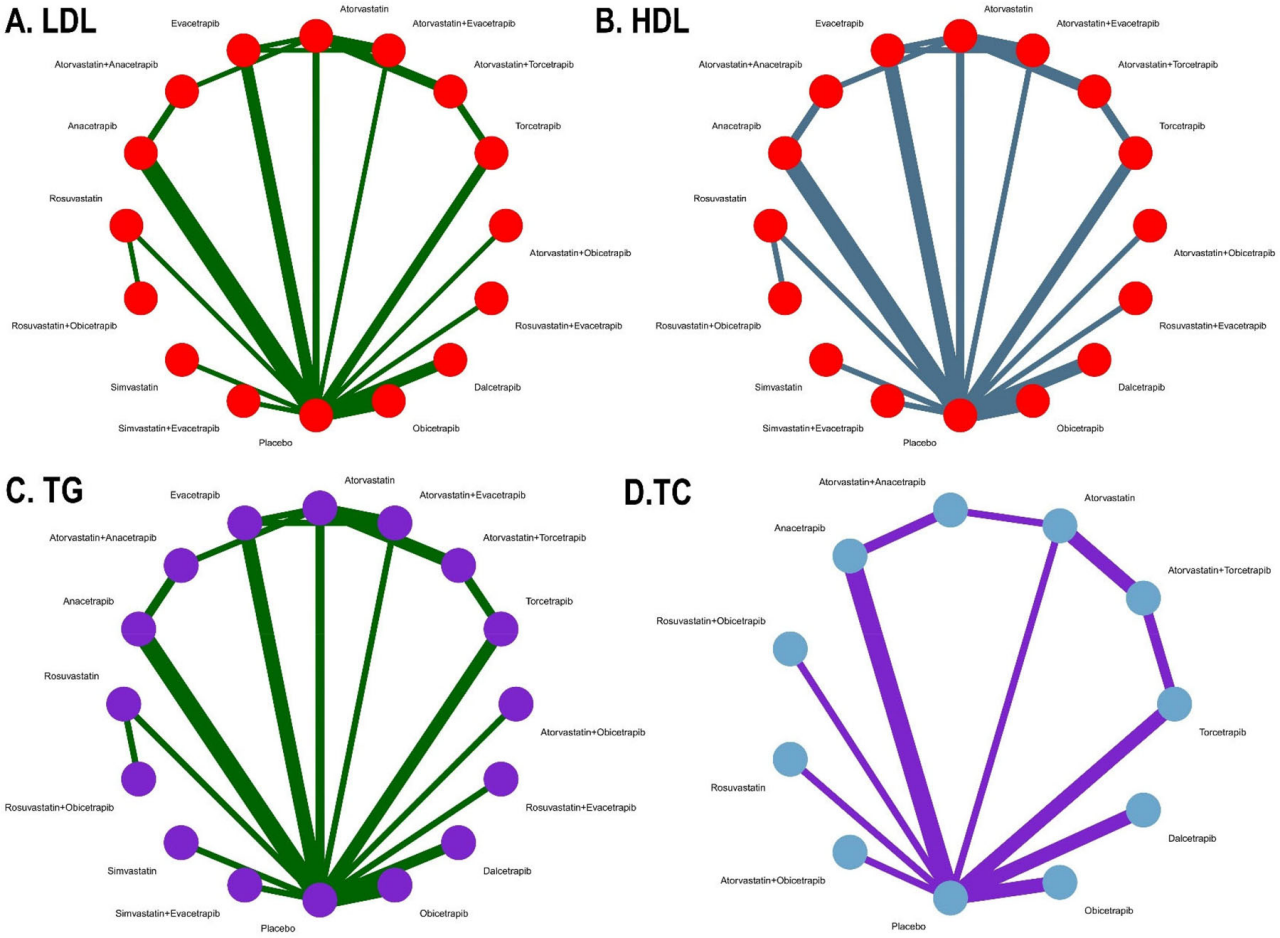
Network plots showing the treatment arms of outcomes of interest of included studies. Outcomes are denoted here as A. Low density lipoprotein (LDL‐C), B. High density lipoprotein (HDL‐C), C. Triglycerides (TG), and D. Total cholesterol (TC).

This summary of baseline characteristics ensures that the network meta‐analysis accounts for demographic and clinical heterogeneity, enhancing the generalizability of findings to clinical practice. The study selection process is shown in Figure 1. Further details on specific study‐level characteristics are available in the supplementary table 1. Network plots shows the treatment arms of outcomes of interest of included studies in Figure 2. Risk of bias plot and publication bias of included studies were shown in Supplementary Figures 1 and 2, respectively.

### 3.2 Low‐Density Lipoprotein Cholesterol (LDL‐C)

**Figure 3:**
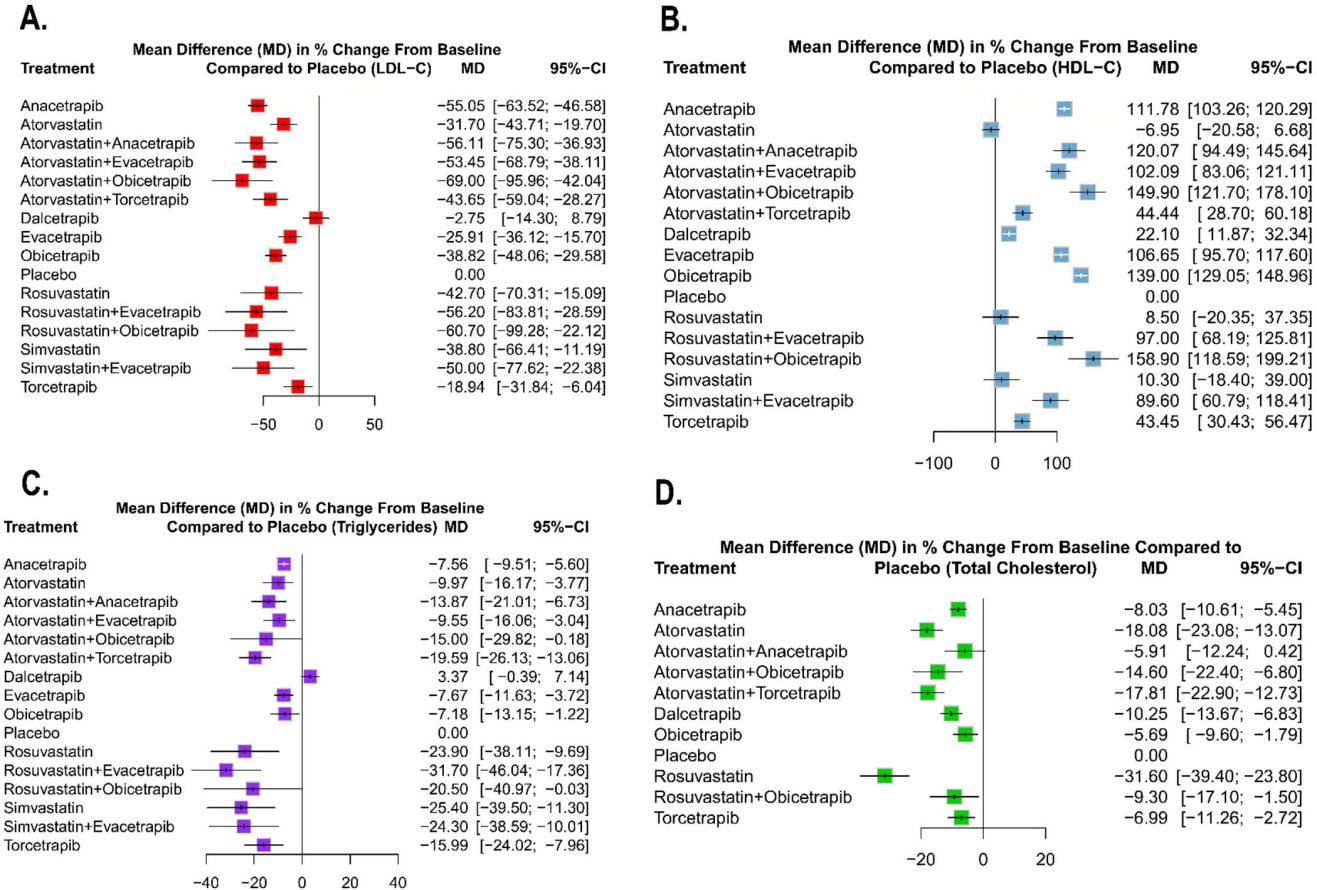
Forest plots showing the mean differences in percentage change from baseline compared to placebo for all the interventions included in this network meta‐analysis. Outcomes are denoted here as A. Low density lipoprotein (LDL‐C), B. High density lipoprotein (HDL‐C), C. Triglycerides (TG), and D. Total cholesterol (TC).

**Figure 4:**
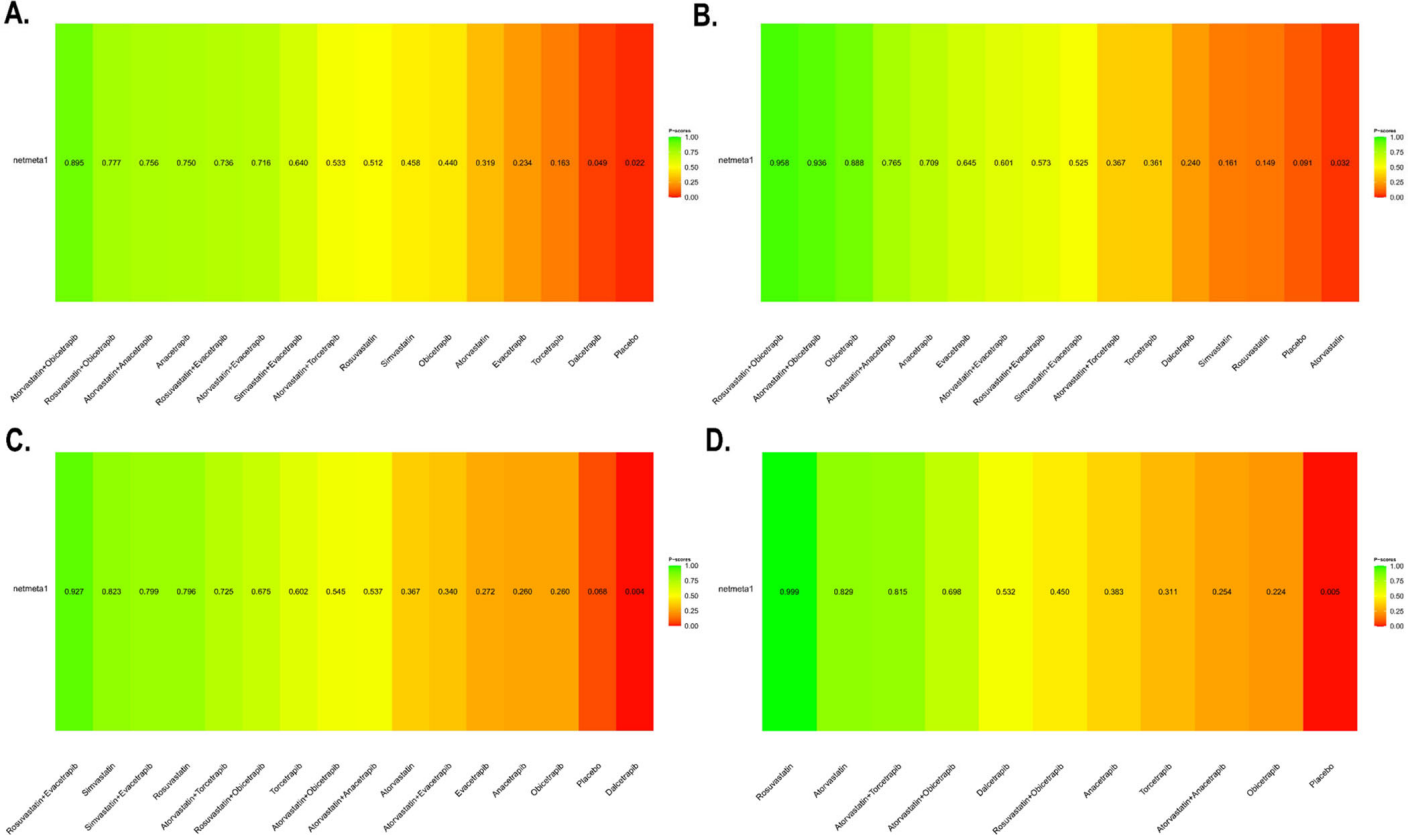
Rank plot showing the ranking of all the interventions included in this network meta‐analysis according to P‐Score; higher P‐Score means higher intervention rankings for respective outcomes. Outcomes are denoted here as A. Low density lipoprotein (LDL‐C), B. High density lipoprotein (HDL‐C), C. Triglycerides (TG), and D. Total cholesterol (TC).

**Figure 5:**
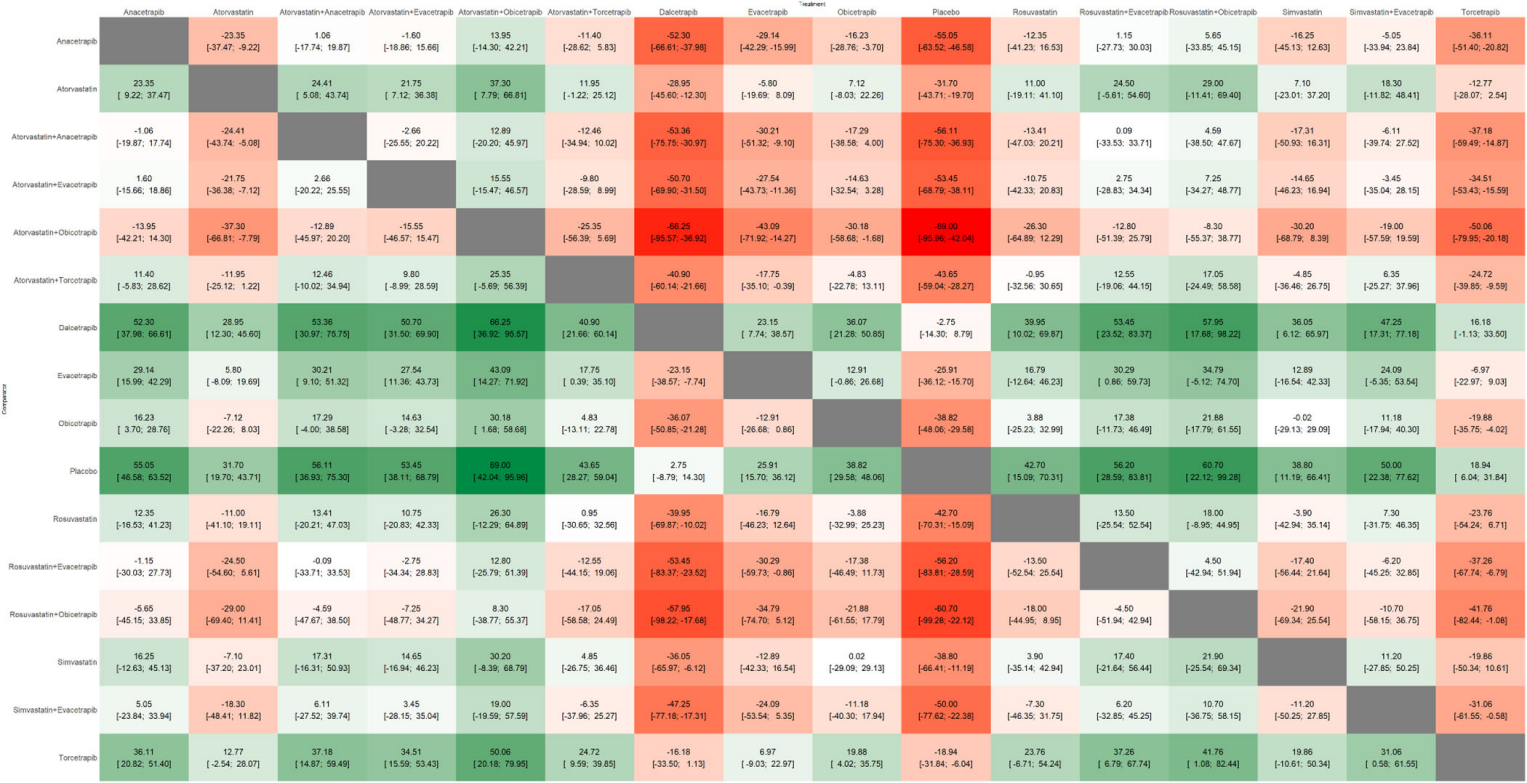
League table with heatmap showing the head‐to‐head comparison among different interventions included in this network meta‐analysis for the outcome of LDL‐C.

In the random‐effects network meta‐analysis evaluating the impact of treatments on LDL‐C levels compared to placebo, several interventions demonstrated significant reductions. A total of 22 studies encompassed 91,382 patients for the outcome LDL‐C with the network plot shown in Figure 2A, forest plot in Figure 3A, intervention ranking plot in Figure 4A, and league table with heatmap in Figure 5. Atorvastatin combined with obicetrapib showed the largest reduction in LDL‐C levels (MD: −69.00, 95% CI: −95.96 to −42.04, *p* < 0.0001), followed by rosuvastatin combined with obicetrapib (MD: −60.70, 95% CI: −99.28 to ‐22.12, *p* = 0.0020). Atorvastatin combined with anacetrapib (MD: −56.11, 95% CI: −75.30 to −36.93, *p* < 0.0001) and rosuvastatin combined with evacetrapib (MD: −56.20, 95% CI: −83.81 to −28.59, *p* < 0.0001) also demonstrated substantial efficacy. Among monotherapies, anacetrapib significantly reduced LDL‐C levels (MD: −55.05, 95% CI: −63.52 to −46.58, *p* < 0.0001), as did obicetrapib (MD: −38.82, 95% CI: −48.06 to −29.58, *p* < 0.0001) and evacetrapib (MD: −25.91, 95% CI: −36.12 to −15.70, *p* < 0.0001). Dalcetrapib did not produce a statistically significant reduction (MD: −2.75, 95% CI: −14.30 to 8.79, *p* = 0.6403).

Statin monotherapies also showed significant LDL‐C reductions, with rosuvastatin (MD: −42.70, 95% CI: −70.31 to −15.09, *p* = 0.0024) and simvastatin (MD: ‐38.80, 95% CI: −66.41 to −11.19, *p* = 0.0059) being effective. Combined regimens such as atorvastatin + evacetrapib (MD: −53.45, 95% CI: −68.79 to ‐−38.11, *p* < 0.0001) and simvastatin + evacetrapib (MD: −50.00, 95% CI: −77.62 to −22.38, *p* = 0.0004) provided additional reductions. Heterogeneity was high (τ² = 180.99; I² = 98.3%, *p* < 0.0001), indicating significant variability across studies. Both within‐design heterogeneity (Q = 2521.91, *p* < 0.0001) and between‐design inconsistency (Q = 214.74, *p* < 0.0001) were significant. P‐scores under the random‐effects model revealed the relative effectiveness of treatments and used to rank the interventions (Figure 4A). Atorvastatin + obicetrapib ranked highest (P‐score: 0.8948), followed by rosuvastatin + obicetrapib (P‐score: 0.7772) and atorvastatin + anacetrapib (P‐score: 0.7556). Anacetrapib alone (P‐score: 0.7498) and rosuvastatin + evacetrapib (P‐score: 0.7361) were also effective. Placebo had the lowest score (P‐score: 0.0218), confirming minimal effectiveness.

### 3.3 High‐Density Lipoprotein Cholesterol (HDL‐C)

**Figure 6:**
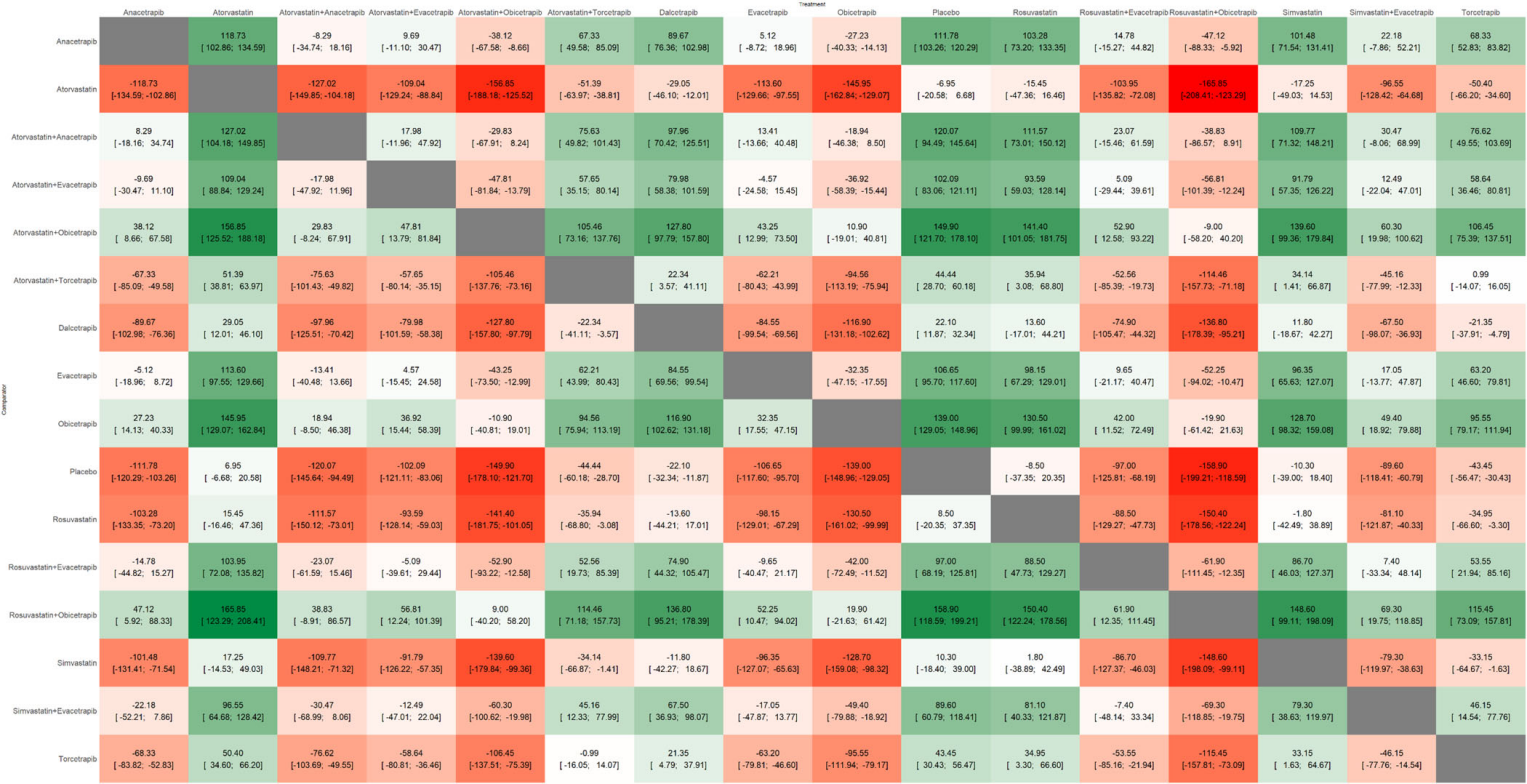
League table with heatmap showing the head‐to‐head comparison among different interventions included in this network meta‐analysis for the outcome of HDL‐C.

A total of 21 studies encompassed 90,991 patients for the outcome HDL‐C with the network plot shown in Figure 2B, forest plot in Figure 3B, intervention ranking plot in Figure 4B, and league table with heatmap in Figure 6. In the random‐effects network meta‐analysis evaluating the effects of treatments on HDL‐C levels compared to placebo, several interventions showed significant increases. Among the treatments, atorvastatin combined with obicetrapib yielded highly significant increase in HDL‐C levels (MD: 149.90, 95% CI: 121.70 to 178.10, *p* < 0.0001), but the greatest increase showed by rosuvastatin combined with obicetrapib (MD: 158.90, 95% CI: 118.59 to 199.21, *p* < 0.0001) and obicetrapib monotherapy (MD: 139.00, 95% CI: 129.05 to 148.96, *p* < 0.0001).

Combination therapies such as atorvastatin + anacetrapib (MD: 120.07, 95% CI: 94.49 to 145.64, *p* < 0.0001) and atorvastatin + evacetrapib (MD: 102.09, 95% CI: 83.06 to 121.11, *p* < 0.0001) also demonstrated substantial efficacy. Among CETP inhibitors, anacetrapib (MD: 111.78, 95% CI: 103.26 to 120.29, *p* < 0.0001) and evacetrapib (MD: 106.65, 95% CI: 95.70 to 117.60, *p* < 0.0001) were effective in increasing HDL‐C levels. Dalcetrapib (MD: 22.10, 95% CI: 11.87 to 32.34, *p* < 0.0001) and torcetrapib (MD: 43.45, 95% CI: 30.43 to 56.47, *p* < 0.0001) achieved modest improvements.

Statins alone were less effective, with rosuvastatin (MD: 8.50, 95% CI: −20.35 to 37.35, *p* = 0.5636), simvastatin (MD: 10.30, 95% CI: −18.40 to 39.00, *p* = 0.4818), and atorvastatin (MD: −6.95, 95% CI: −20.58 to 6.68, *p* = 0.3178) showing minimal or nonsignificant effects. Combined regimens, including simvastatin + evacetrapib (MD: 89.60, 95% CI: 60.79 to 118.41, *p* < 0.0001) and rosuvastatin + evacetrapib (MD: 97.00, 95% CI: 68.19 to 125.81, *p* < 0.0001), were more effective. P‐scores under the random‐effects model indicated the relative effectiveness of treatments (Figure 4B). Rosuvastatin + obicetrapib achieved the highest P‐score (P‐score: 0.9583), followed by atorvastatin + obicetrapib (P‐score: 0.9361) and obicetrapib monotherapy (P‐score: 0.8879). Atorvastatin + anacetrapib (P‐score: 0.7647) and anacetrapib alone (P‐score: 0.7088) also ranked highly.

### 3.4 Triglycerides

**Figure 7:**
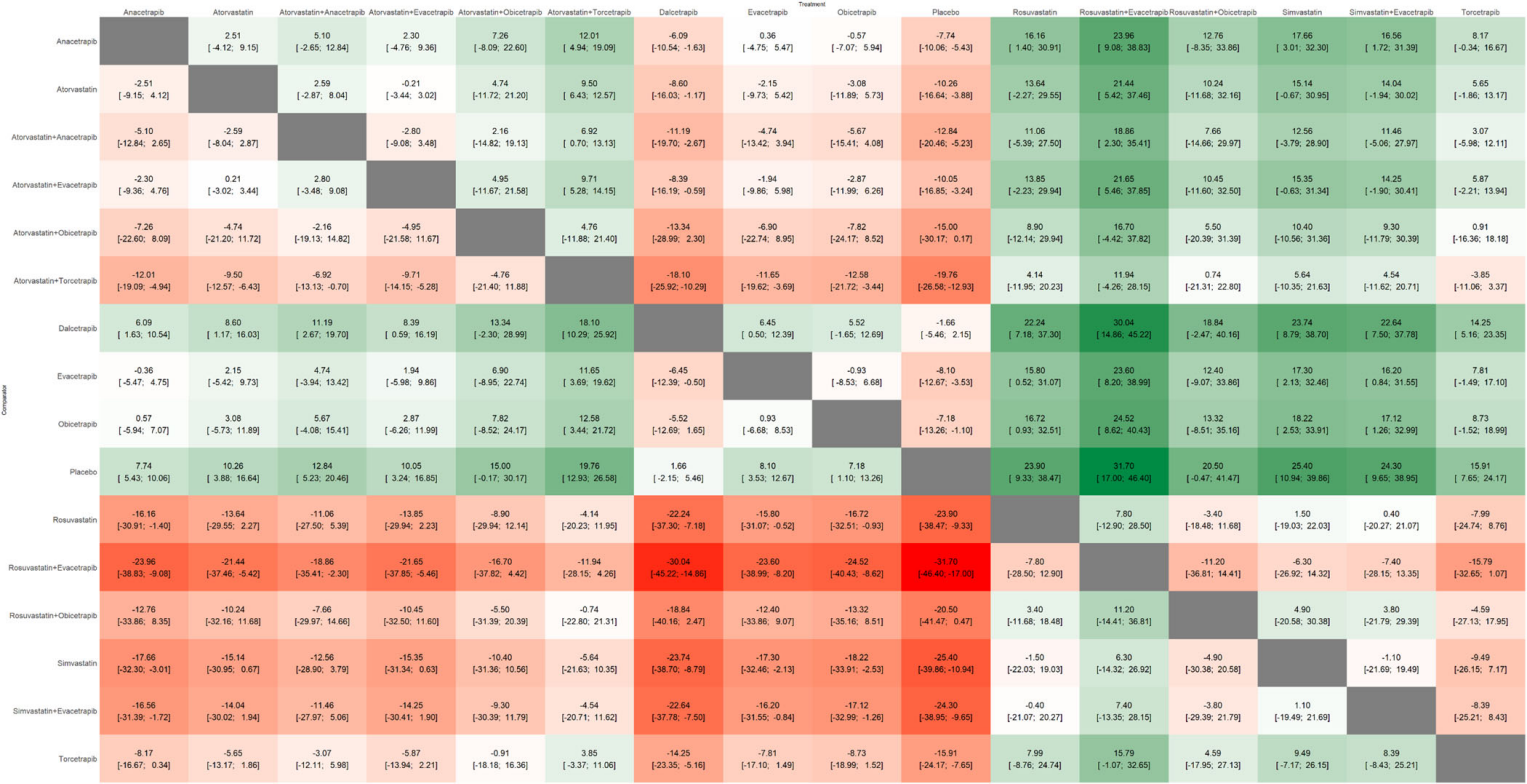
League table with heatmap showing the head‐to‐head comparison among different interventions included in this network meta‐analysis for the outcome of triglycerides (TG).

A total of 18 studies encompassed 55,973 patients for this outcome, with the network plot shown in Figure 2C, forest plot in Figure 3C, intervention ranking plot in Figure 4C, and league table with heatmap in Figure 7. In the random‐effects network meta‐analysis evaluating the effects of treatments on triglyceride levels compared to placebo, several therapies demonstrated significant reductions. The combination of rosuvastatin + evacetrapib showed the largest reduction in triglycerides (MD: −31.70, 95% CI: −46.04 to −17.36, *p* < 0.0001), followed by simvastatin (MD: −25.40, 95% CI: −39.50 to −11.30, *p* = 0.0004) and simvastatin + evacetrapib (MD: −24.30, 95% CI: −38.59 to −10.01, *p* = 0.0009). Rosuvastatin alone (MD: −23.90, 95% CI: −38.11 to −9.69, *p* = 0.0010) also showed a substantial reduction.

Among combination therapies, atorvastatin + torcetrapib (MD: −19.59, 95% CI: −26.13 to −13.06, *p* < 0.0001) and rosuvastatin + obicetrapib (MD: −20.50, 95% CI: −40.97 to −0.03, *p* = 0.0496) demonstrated significant reductions. Torcetrapib alone (MD: −15.99, 95% CI: −24.02 to −7.96, *p* < 0.0001) and atorvastatin + anacetrapib (MD: −13.87, 95% CI: −21.01 to −6.73, *p* = 0.0001) were moderately effective. Atorvastatin + obicetrapib (MD: −15.00, 95% CI: −29.82 to −0.18, *p* = 0.0473) and atorvastatin + evacetrapib (MD: −9.55, 95% CI: −16.06 to −3.04, *p* = 0.0040) also showed reductions. Among CETP inhibitors, evacetrapib (MD: −7.67, 95% CI: −11.63 to −3.72, *p* = 0.0001) and anacetrapib (MD: −7.56, 95% CI: −9.51 to ‐5.60, *p* < 0.0001) were effective in reducing triglycerides. Obicetrapib demonstrated a smaller reduction (MD: −7.18, 95% CI: −13.15 to −1.22, *p* = 0.0183). Dalcetrapib, however, did not result in a statistically significant reduction (MD: 3.38, 95% CI: −0.39 to 7.14, *p* = 0.0790). P‐scores under the random‐effects model (Figure 4C) ranked rosuvastatin + evacetrapib as the most effective treatment (P‐score: 0.9269), followed by simvastatin (P‐score: 0.8235) and simvastatin + evacetrapib (P‐score: 0.7993). Rosuvastatin alone also ranked highly (P‐score: 0.7955). Moderate P‐scores were noted for atorvastatin + torcetrapib (P‐score: 0.7249), rosuvastatin + obicetrapib (P‐score: 0.6754), and torcetrapib (P‐score: 0.6018).

### 3.5 Total Cholesterol

**Figure 8:**
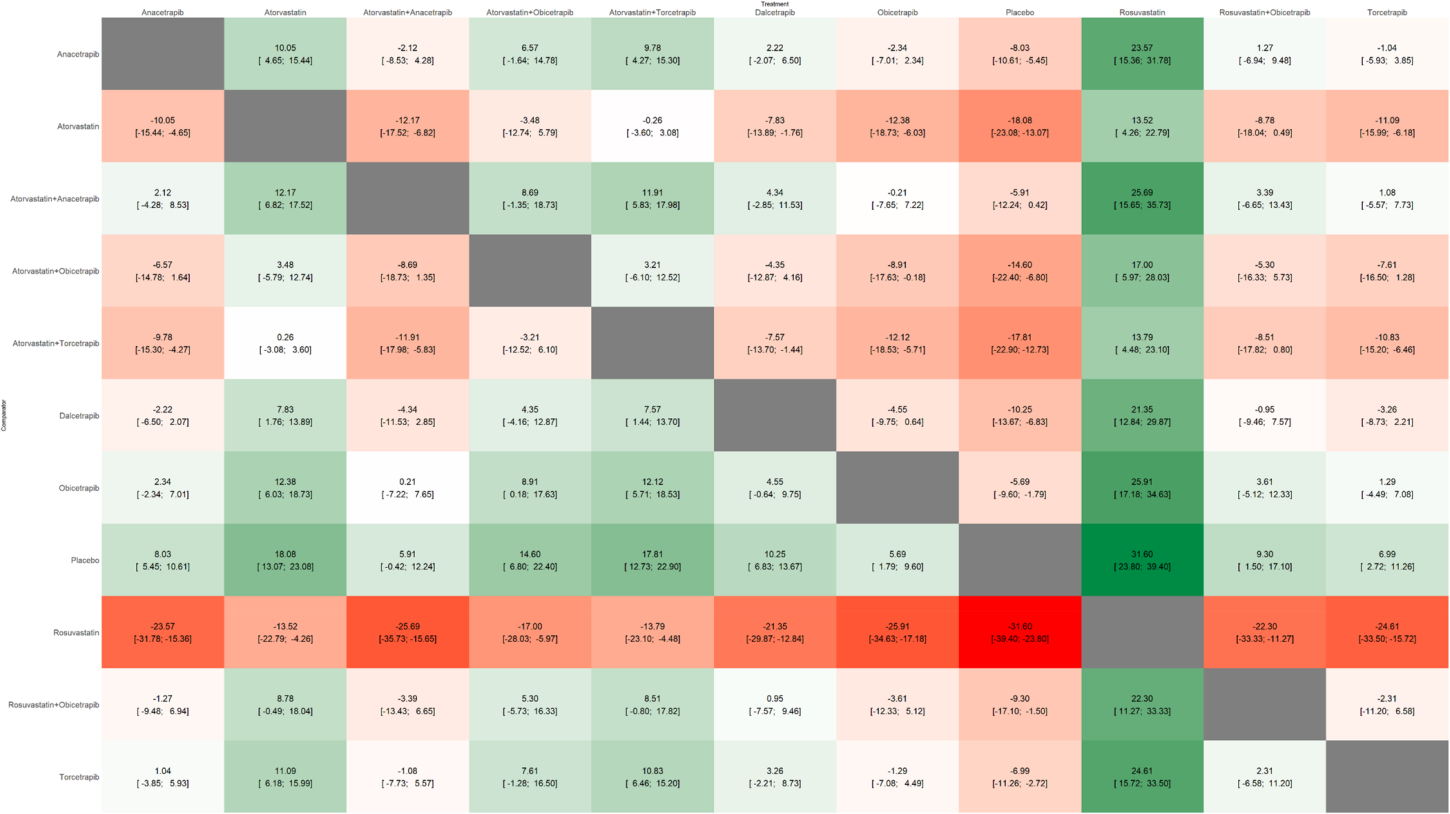
League table with heatmap showing the head‐to‐head comparison among different interventions included in this network meta‐analysis for the outcome of total cholesterol (TC).

A total of 13 studies encompassed 42,755 patients for this outcome with the network plot shown in Figure 2D, forest plot in Figure 3D, intervention ranking plot in Figure 4D, and league table with heatmap in Figure 8. In the random‐effects network meta‐analysis assessing the effects of various treatments on total cholesterol levels compared to placebo, several therapies demonstrated significant reductions. Rosuvastatin exhibited the greatest reduction in total cholesterol levels (MD: −31.60, 95% CI: −39.40 to −23.80, *p* < 0.0001), followed by atorvastatin (MD: −18.08, 95% CI: −23.08 to −13.07, *p* < 0.0001) and atorvastatin + torcetrapib (MD: −17.81, 95% CI: −22.90 to −12.73, *p* < 0.0001). Combination therapies, including atorvastatin + obicetrapib (MD: −14.60, 95% CI: −22.40 to −6.80, *p* = 0.0002), were also effective.

Among CETP inhibitors, dalcetrapib (MD: −10.25, 95% CI: −13.67 to −6.83, *p* < 0.0001) and anacetrapib (MD: −8.03, 95% CI: −10.61 to −5.45, *p* < 0.0001) showed significant reductions, while torcetrapib (MD: −6.99, 95% CI: −11.26 to −2.72, *p* = 0.0013) and obicetrapib (MD: −5.69, 95% CI: −9.60 to −1.79, *p* = 0.0043) provided modest improvements. The combination of rosuvastatin + obicetrapib resulted in a smaller but still statistically significant reduction (MD: −9.30, 95% CI: −17.10 to −1.50, *p* = 0.0194). In contrast, atorvastatin + anacetrapib (MD: −5.91, 95% CI: −12.24 to 0.42, *p* = 0.0672) did not achieve statistical significance. P‐scores under the random‐effects model highlighted the relative effectiveness of treatments (Figure 4D). Rosuvastatin achieved the highest P‐score (P‐score: 0.999), followed by atorvastatin (P‐score: 0.829) and atorvastatin + torcetrapib (P‐score: 0.815). Moderate P‐scores were observed for atorvastatin + obicetrapib (P‐score: 0.698) and dalcetrapib (P‐score: 0.532).

### 3.6 Sensitivity Analysis

For the sensitivity analysis of all outcomes, we employed multiple visualization techniques to assess the robustness and reliability of the results. First, we generated a heatmap to visually represent the effect sizes of various treatment comparisons, using a color gradient to highlight the magnitude of effects on lipid parameters such as LDL‐C, HDL‐C, and all other outcomes (Supplementary Figure 3‐6). This allowed for easy identification of significant treatment effects and relationships between interventions. Additionally, we created a heatplot to explore pairwise treatment comparisons and further examine correlations between different interventions and their effects. To complement these visualizations, we also used a splited forest plot to assess the impact of each individual study on the overall results, providing a clear view of study‐level variability (Supplementary Figures 7–10). Together, these analyses allowed us to identify potential sources of heterogeneity, evaluate the consistency of treatment effects, and ensure the robustness of the conclusions drawn from the network meta‐analysis.

## 4 Discussion

Hyperlipidemia remains one of the most critical modifiable risk factors for cardiovascular disease (CVD). Elevated levels of low‐density lipoprotein cholesterol (LDL‐C), alongside increased triglycerides and reduced high‐density lipoprotein cholesterol (HDL‐C), are pivotal in the development of atherosclerosis, which, in turn, underlies most cardiovascular events [18, 19]. Current lipid‐lowering therapies, including statins and fibrates, primarily target LDL‐C and triglycerides, but there is a growing need to address the lower levels of HDL‐C, as this is associated with reverse cholesterol transport, a protective mechanism against atherosclerosis. CETP inhibitors (such as anacetrapib, obicetrapib, evacetrapib, and dalacetrapib) have been identified as promising candidates due to their potential to increase HDL‐C while simultaneously reducing LDL‐C, total cholesterol, and triglycerides levels [20, 21, 22, 23]. However, while previous trials have shown variability in their outcomes, the comparative effectiveness of these drugs remains poorly defined. Our study provides a comprehensive evaluation of these therapies using a network meta‐analysis, comparing their effectiveness on key lipid parameters across multiple randomized controlled trials (RCTs). This network meta‐analysis provides a comprehensive evaluation of lipid‐lowering therapies, incorporating 33 randomized controlled trials (RCTs) with 120,292 participants. The findings highlight the superior efficacy of combination therapies, particularly atorvastatin + obicetrapib and rosuvastatin + obicetrapib, in reducing LDL‐C and increasing HDL‐C, while monotherapies such as anacetrapib and evacetrapib also demonstrated significant effects. Rosuvastatin alone was the most effective in lowering total cholesterol, and rosuvastatin + evacetrapib led in triglyceride reduction. The analysis includes a diverse range of interventions, covering statins (atorvastatin, rosuvastatin, simvastatin), and CETP inhibitors (anacetrapib, torcetrapib, evacetrapib, obicetrapib, dalcetrapib), across different doses and treatment durations.

The high quality of evidence in this network meta‐analysis is supported by the inclusion of RCTs with rigorous methodologies, such as randomization, allocation concealment, and blinding, ensuring reliable comparisons. The large sample size enhances statistical power, while the network meta‐analysis framework enables an integrated evaluation of multiple interventions, allowing for a comprehensive ranking of therapies based on efficacy. While heterogeneity was present, the overall treatment rankings remained consistent across different analyses, and did not change significantly during sensitivity analysis, reinforcing the reliability of the findings and providing valuable insights into the comparative effectiveness of these therapies.

### 4.1 CETP Inhibitors: Mechanisms and Efficacy

Cholesteryl ester transfer protein (CETP) inhibitors have emerged as a promising class of lipid‐lowering agents due to their ability to modify lipid profiles through the inhibition of CETP. By preventing the transfer of cholesteryl esters from HDL to atherogenic lipoproteins such as LDL and VLDL, CETP inhibitors help preserve HDL‐C levels while reducing the atherogenic lipoproteins that contribute to the formation of plaques in the arteries [24, 25, 26]. The results from this study corroborate previous findings from large‐scale trials on CETP inhibitors, such as the DEFINE trial (Brinton et al., 2014), ILLUMINATE trial (Barter et. Al, 2007), and HPS3/TIMI55‐REVEAL trial (Bowman et al., 2017), which demonstrated a significant reduction in LDL‐C and an increase in HDL‐C with anacetrapib [27, 28, 29]. Our study shows that obicetrapib, as a CETP inhibitor, achieved similar outcomes, reducing LDL‐C by 38.82% (MD: −38.82, 95% CI: −48.06 to ‐29.58) and significantly increasing HDL‐C by 139.00% (MD, 95% CI: 129.05 to 148.96).

Further comparison with the Phase II trial of obicetrapib (Harada‐Shiba et al., 2024), which reported a 159% increase in HDL‐C, highlights the consistency of obicetrapib's effects in raising HDL‐C levels [30]. In our study, the combination of atorvastatin + obicetrapib yielded an increase in HDL‐C (MD: 149.90%, 95% CI: 121.70 to 178.10), supporting obicetrapib's role as an effective adjunctive therapy. These results are further in line with the TULIP study (Hovingh et al., 2015), where obicetrapib demonstrated significant HDL‐C elevation of more than 150% from baseline [31], reinforcing the importance of CETP inhibitors in improving lipid profiles and offering potential therapeutic benefits, particularly in combination with statins.

### 4.2 LDL‐C Reduction: A Critical Endpoint in Cardiovascular Risk Reduction

One of the most important outcomes for lipid‐lowering therapy is the reduction of LDL‐C, which has been consistently linked to decreased cardiovascular events. Our results show that combination therapies involving statins and CETP inhibitors provide substantial reductions in LDL‐C, far exceeding the reductions achieved by monotherapies. Specifically, atorvastatin combined with obicetrapib led to the largest reduction in LDL‐C (MD: −69.00, 95% CI: −95.96 to −42.04, *p* < 0.0001), followed by rosuvastatin combined with obicetrapib (MD: −60.70, 95% CI: −99.28 to −22.12, *p* = 0.0020), which highlights the superior efficacy of CETP inhibitors in combination with statins. These findings corroborate with the DEFINE trial (Brinton et al., 2015), and other trials, which demonstrated that anacetrapib (an earlier‐generation CETP inhibitor) achieved a reduction in LDL‐C by 40%, underlining the value of CETP inhibition as a viable treatment strategy [27, 32, 33, 34].

On the other hand, CETP inhibitors as monotherapies also showed significant efficacy. Anacetrapib (MD: −55.05, 95% CI: −63.52 to −46.58, *p* < 0.0001) and obicetrapib (MD: −38.82, 95% CI: −48.06 to −29.58, *p* < 0.0001) were particularly effective in reducing LDL‐C levels, aligning with the results from previous studies, such as the few trial for anacetrapib, where it demonstrated significant reductions in LDL‐C. Evacetrapib (MD: −25.91, 95% CI: −36.12 to −15.70, *p* < 0.0001) also showed a meaningful decrease in LDL‐C, but to a lesser extent, aligning with previous trials like the ACCELERATE study (Lincoff et al., 2017), which demonstrated that evacetrapib effectively reduced LDL‐C but less robustly compared to other CETP inhibitors [35, 36, 37, 38]. Interestingly, dalacetrapib did not produce a statistically significant reduction in LDL‐C (MD: −2.75, 95% CI: −14.30 to 8.79, *p* = 0.6403), consistent with findings from the dal‐OUTCOMES trial (Schwartz et al., 2017), where dalacetrapib showed limited efficacy, providing a stark contrast to the more potent effects seen with anacetrapib and obicetrapib [39, 40].

### 4.3 HDL‐C Elevation: The Promising Role of CETP Inhibitors

In contrast to statins, which have a limited ability to raise HDL‐C, CETP inhibitors are designed to increase HDL‐C by preventing the transfer of cholesterol esters to atherogenic lipoproteins [1, 3]. Our results showed that combination therapies were superior in elevating HDL‐C levels. Atorvastatin combined with obicetrapib resulted in the second largest increase in HDL‐C (MD: 149.90, 95% CI: 121.70 to 178.10, *p* < 0.0001), while greatest increase shown by rosuvastatin combined with obicetrapib (MD: 158.90, 95% CI: 118.59 to 199.21, *p* < 0.0001), highlighting the additive benefit of combining CETP inhibitors with statins. These findings are in line with the results from the TULIP trial (Hovingh et al., 2015), where obicetrapib significantly increased HDL‐C [31], further supporting the potential of CETP inhibitors in raising HDL‐C to levels that could be clinically beneficial in reducing cardiovascular risk.

Obicetrapib as monotherapy also demonstrated substantial efficacy in increasing HDL‐C (MD: 139.00, 95% CI: 129.05 to 148.96, *p* < 0.0001), which aligns with the findings from Harada‐Shiba et al. (2024), where obicetrapib was shown to produce a 159% increase in HDL‐C [30]. This effect is particularly important as higher HDL‐C levels are linked to reduced cardiovascular risk. Conversely, statins alone were less effective in increasing HDL‐C, with rosuvastatin (MD: 8.50, 95% CI: −20.35 to 37.35, *p* = 0.5636) showing only a marginal effect, consistent with the findings from Schwartz et al. (2020), and other trials where statins did not significantly alter HDL‐C levels [39, 41].

### 4.4 Total Cholesterol and Triglycerides: Modulation of Lipid Profiles

In terms of total cholesterol reduction, our meta‐analysis showed that rosuvastatin was the most effective (MD: −31.60, 95% CI: −39.40 to −23.80, *p* < 0.0001), closely followed by atorvastatin (MD: −18.08, 95% CI: −23.08 to −13.07, *p* < 0.0001), which is consistent with findings from trials such as the JUPITER study (Ridker et al., 2008) [42]. Combination therapies, including rosuvastatin and obicetrapib (MD: −9.30, 95% CI: −17.10 to −1.50, *p* = 0.0194), also showed modest reductions in total cholesterol, supporting the potential of CETP inhibitors in conjunction with statins to further optimize lipid profiles.

For triglycerides, rosuvastatin combined with evacetrapib produced the greatest reduction (MD: −31.70, 95% CI: −46.04 to −17.36, *p* < 0.0001), followed by simvastatin (MD: −25.40, 95% CI: −39.50 to −11.30, *p* = 0.0004). These findings align with previous studies like the ACCELERATE trial (Lincoff et al., 2017) where evacetrapib led to significant reductions in triglycerides, indicating its robust effects in addressing this lipid parameter [35].

Monotherapies with CETP inhibitors like anacetrapib (MD: −7.56, 95% CI: −9.51 to −5.60, *p* < 0.0001) and evacetrapib (MD: −7.67, 95% CI: −11.63 to −3.72, *p* = 0.0001) also demonstrated effective reductions in triglycerides, supporting their potential role in managing lipid imbalances. Dalcetrapib, however, did not significantly reduce triglycerides (MD: 3.38, 95% CI: −0.39 to 7.14, *p* = 0.0790), in line with the findings of the dal‐OUTCOMES trial (Schwartz et al., 2017), and other trials where dalacetrapib showed limited effects on triglycerides [39, 43, 44].

### 4.5 Clinical Implications and Comparative Effectiveness of Combination versus Monotherapy

Our findings highlight the substantial benefits of combining CETP inhibitors with statins, particularly in improving HDL‐C, LDL‐C, and triglycerides, thereby optimizing lipid management. Combination therapies like atorvastatin + obicetrapib and rosuvastatin + obicetrapib were found to be more effective in modulating lipid profiles compared to monotherapies, which suggests a synergistic effect when these drugs are used together [45, 46, 47]. These results align with previous trials, such as the ACCELERATE study (Lincoff et al., 2017), and a trial done by Nicholls et al. (2011) which demonstrated that CETP inhibitors in combination with statins provide a comprehensive approach to managing dyslipidemia [35, 37].

Monotherapy with CETP inhibitors like anacetrapib and obicetrapib also showed considerable efficacy, particularly in LDL‐C reduction and HDL‐C elevation [37, 44, 47]. However, when combined with statins, these agents produced superior results as demonstrated in our network meta‐analysis, reinforcing the need for dual‐therapy strategies to address multiple lipid parameters simultaneously. The observed superior efficacy of combination therapies may reduce the need for other adjunct therapies, making them an attractive option for managing complex lipid imbalances in high‐risk cardiovascular patients [48, 49, 50].

### 4.6 Study Limitations and Future Directions

While this study provides valuable insights into the comparative effectiveness of CETP inhibitors, several limitations should be considered. Firstly, our analysis primarily focused on lipid profile changes, rather than direct clinical outcomes such as cardiovascular events (e.g., myocardial infarction, stroke, or mortality). While changes in lipid levels are critical indicators of cardiovascular risk, the ultimate goal of lipid‐lowering therapies is to reduce these events. Therefore, future studies should focus on long‐term follow‐up to evaluate the impact of CETP inhibitors on actual cardiovascular outcomes, as long‐term effects of these drugs remain to be elucidated [51, 52, 53].

Secondly, significant heterogeneity across studies was observed, especially in study design, treatment regimens, and follow‐up durations. Variability in dosing regimens and patient populations may influence the generalizability of these findings. The high I² statistic (98.3%) suggests that the treatment effects may differ across patient subgroups, particularly based on comorbidities or adherence patterns. Sensitivity analyses should be conducted in future trials to assess the robustness of CETP inhibitors across diverse populations.

Finally, while CETP inhibitors such as obicetrapib and anacetrapib demonstrated promising pharmacokinetic profiles with minimal accumulation and rapid elimination, the long‐term safety of these therapies remains uncertain [49]. Future research should focus on assessing the risk of off‐target effects, including potential cardiovascular events and other adverse effects, particularly in real‐world settings.

## 5 Conclusion

In conclusion, this network meta‐analysis demonstrates that **CETP inhibitors**, particularly in combination with **statins**, are highly effective in improving lipid profiles, including **LDL‐C**, **HDL‐C**, **triglycerides**, and **total cholesterol**. The superiority of combination therapies compared to monotherapies highlights the potential of these drugs to provide a comprehensive approach to managing dyslipidemia in high‐risk cardiovascular patients. However, further studies are required to evaluate the long‐term clinical outcomes and safety of these therapies, particularly in diverse populations. The findings underscore the importance of **CETP inhibitors** as promising candidates for reducing residual cardiovascular risk and optimizing lipid management. Combining real‐world data with clinical trial results will be essential in bridging the gap between lipid profile changes and actual cardiovascular benefits.

## Conflicts of Interest

The authors declare no conflicts of interest.

## Ethics Statement

Ethical review and approval were waived for this meta‐analysis due to the use of available aggregated data from the included trials. This study adhered to ethical guidelines for secondary data analysis, with no direct involvement of human participants.

## Supporting information


**Supplementary Table 1:** Population Baseline Characteristics of Included Studies with References.

**Figure S1:** Plot showing the risk of bias assessment (ROB2) of included studies. **Figure S2:** Funnel plot with Egger test showing the publication bias of included studies. **Figure S3:** Heatmap showing the heterogeneity and inconsistency results of interventions of included studies for the outcome LDL‐C. **Figure S4:** Heatmap showing the heterogeneity and inconsistency results of interventions of included studies for the outcome HDL‐C. **Figure S5:** Heatmap showing the heterogeneity and inconsistency results of interventions of included studies for the outcome TG. **Figure S6:** Heatmap showing the heterogeneity and inconsistency results of interventions of included studies for the outcome TC. **Figure S7:** Splitted forest plot showing differences among direct and indirect comparisons of intervention for the outcome LDL‐C. **Figure S8:** Splitted forest plot showing differences among direct and indirect comparisons of intervention for the outcome HDL‐C. **Figure S9:** Splitted forest plot showing differences among direct and indirect comparisons of intervention for the outcome TG. **Figure S10:** Splitted forest plot showing differences among direct and indirect comparisons of intervention for the outcome TC.

## Data Availability

The data analyzed for this meta‐analysis are available from the individual included trials and can be accessed upon request.
