# Supplementary material for: Comparative Effectiveness of Cholesteryl Ester Transfer Protein (CETP) Inhibitors on Lipid Profiles in Adults With Hyperlipidemia: A Comprehensive Systematic Review and Frequentist Network Meta‐Analysis of Randomized Controlled Trials
Source: Clin Cardiol. 2025 Sep 14;48(9):e70204. doi: 10.1002/clc.70204 (PMC12434180; doi:10.1002/clc.70204)
Supplement: Supplementary file 1 — Supplementary Table 1: Population Baseline Characteristics of Included Studies with References. [file CLC-48-e70204-s001.docx]

| Study | Intervention name | Population in the group | Mean Age with SD | Sex (Male %) | Baseline Mean LDLC Level and SD | Baseline Mean HDLC Level and SD | Baseline Mean Total Cholersterol Level and SD | Baseline Mean Triglyerides Level and SD |
| --- | --- | --- | --- | --- | --- | --- | --- | --- |
| Mariko Harada-Shiba et.al,2024[1] | Placebo | 26 | 63.3(9.44) | 76.9 | 110(37.1) | 53.2(14.6) | NA | 126(45.5) |
|  | Obicetrapid 2.5 mg | 25 | 65.8(8.09) | 60 | 102(26.6) | 55.6(17.5) | NA | 148(106) |
|  | Obicetrapid 5 mg | 25 | 67.8(9.46) | 76 | 106(27.5) | 53.0(11.3) | NA | 154(71.9) |
|  | Obicetrapid 10 mg | 26 | 62.5(9.90) | 73.1 | 109(32.5) | 54.9(14.2) | NA | 126(52.5) |
| John S. Millar et.al,2016[2] | Anacetrapid 20mg | 29 | 47(10) | 69 | 140(27) | 50(14) | 216(32) | 114.33(45.22) |
|  | Placebo | 10 | 51(11) | 60 | 131(20) | 46(13) | 207(27) | 135(73.10) |
| Benoit J.Arsenault et.al,2017[3] | Atorvastatin and Placebo | 1151 | 62(7.5) | 83 | 77(20) | 46(11) | 153(26) | 151(75) |
|  | Atorvastatin and Torcetrapid | 594 | 62(7.6) | 85 | 77(21) | 47(12) | 153(27) | 146(78) |
| Philip J.Barter et.al,2007[4] | Atorvastatin | 7534 | 61.3(7.6) | 77.8 | 79.9(20.4) | 48.5(12.2) | 157.3(26.9) | 133.33(63.76) |
|  | Torcetrapid plus Atorvastatin | 7533 | 61.3(7.6) | 77.7 | 79.7(20.4) | 48.6(12.0) | 156.8(26.6) | 132.0(63.02) |
| Daniel Bloomfield et.al,2009[5] | Anacetrapid 300mg + Atorvastatin 20mg | 252 | 56.4(9.6) | 42.8 | 1.0(0.12) | 50.5(12.6) | 225.3(28.1) | 153.5(87.0) |
| Eliot A. Brinton et.al,2015[6] | Anacetrapid 100mg | 811 | 62.5(8.7) | 77.6 | 81.4(21.2) | 40.5(9.3) | Na | 127(69.8) |
|  | Placebo | 812 | 62.9(9.0) | 76.1 | 82.2(20.8) | 40.4(9.1) | NA | 129(77.2) |
| Christopher P. Cannon et.al,2010[7] | Anacetrapid | 811 | 62.5(8.7) | 77.6 | 81.4(21.3) | 40.5(9.3) | NA | NA |
|  | Placebo | 812 | 62.9(9.0) | 76.1 | 82.2(20.7) | 40.4(9.1) | NA | NA |
| Michael H. Davidson et.al,2006[8] | placebo | 32 | 47(10) | 78 | 125(31) | 39(7) | 201(36) | 186(84) |
|  | Torcetrapid 10mg/dl | 32 | 48(12) | 69 | 128(28) | 40(5) | 202(35) | 176(63) |
|  | Torcetrapid 30mg/dl | 31 | 45(10) | 84 | 117(27) | 37(5) | 194(32) | 205(88) |
|  | Torcetrapid 60mg/dl | 34 | 46(10) | 79 | 120(22) | 37(6) | 194(29) | 192(88) |
|  | Torcetrapid 90mg/dl | 33 | 49(11) | 82 | 127(29) | 37(6) | 204(30) | 197(78) |
| Gregory G. Schwartz et.al,2020[9] | Normoglycemia (Dalcetrapib) | 1,932 | 58.6(8.7) | 82.9 | 75.3(24.1) | 44.2(12.6) | 143.9(30.6) | 123(69) |
|  | Normoglycemia(Placebo) | 2,018 | 58.6(8.8) | 84.2 | 74.7(24.8) | 43.4(12.2) | 142.4(31.5) | 122(62) |
|  | Prediabetes(Dalcetrapib) | 3,394 | 59.9(9.1) | 80.7 | 78.8(27.1) | 43.0(11.4) | 148.0(33.6) | 132(71) |
|  | Prediabetes(Placebo) | 3,301 | 60(9.0) | 81.7 | 78.2(24.9) | 42.7(11.2) | 147.1(31.9) | 132(74) |
|  | Diabetes(Dalcetrapib) | 2,573 | 61.8(9.1) | 77.4 | 74.1(26.8) | 40.6(11.2) | 143.4(34.0) | 145(78) |
|  | Diabetes(Placebo) | 2,568 | 61.4(9.2) | 78 | 73.4(27.6) | 40.5(11.2) | 142.5(33.5) | 143(80) |
| Antonio M. Gotta et.al,2014[10] | Anacetrapib 100 mg with Placebo | 1,623 | 62.7(8.9) | 76.8 | 81.8( 21.0) | 40.4(9.2) | NA | NA |
|  | Placebo | 1,398 | 62.6(8.8) | 77.5 | 82.4(20.6) | 40.5(9.1) | NA | NA |
| Louise Bowman et. al,2017[11] | Anacetrapib | 15225 | 67(8) | 83.9 | 61(15) | 40(10) | NA | NA |
|  | Placebo | 15224 | 67(8) | 83.8 | 61(15) | 40(10) | NA | NA |
| G Kees Hovingh et. al,2015[12] | Placebo | 40 | 64·4(6·6) | 95 | 146.95(19.34) | 50.27(11.60) | 224.29(19.34) | 54.14(23.20) |
|  | Obicetrapib 1 mg | 41 | 65·8(6·3) | 78 | 139.21(23.20) | 50.27(19.34) | 216.55(27.07) | 54.14(19.34) |
|  | Obicetrapib 2·5 mg | 41 | 65·5(6·9) | 81 | 139.21(19.34) | 54.14(11.60) | 216.55(27.07) | 54.14(30.94) |
|  | Obicetrapib 5 mg | 40 | 64·9(7·8) | 88 | 135.34(23.20) | 50.27(7.73) | 216.55(27.07) | 65.74(3.87) |
|  | Obicetrapib 10 mg | 40 | 66(5·0) | 78 | 135.34(23.20) | 50.27(11.60) | 220.42(23.20) | 65.74(27.07) |
|  | 20 mg Atorvastatin | 40 | 64(9·4) | 70 | 146.95(15.47) | 54.14(7.73) | 228.15(19.34) | 54.14(19.34) |
|  | 20 mg atorvastatin plus 10 mg Obicetrapib | 40 | 63·3(9·1) | 88 | 135.34(23.20) | 50.27(11.60) | 212.69(27.07) | 50.27(23.20) |
|  | 10 mg rosuvastatin | 41 | 64·7(8·0) | 73 | 143.08(23.20) | 50.27(11.60) | 224.29(27.07) | 61.87(27.07) |
|  | 10 mg rosuvastatin plus 10 mg Obicetrapib | 41 | 63·8(6·1) | 85 | 139.21(23.20) | 54.14(11.60) | 216.55(27.07) | 54.14(23.20) |
| John J.P. Kastelein et. al,2007[13] | Atorvastatin monotherapy | 454 | 45.2(12.9) | 51.1 | 138.9(37.6) | 51.8(12.8) | 213.5(42.1) | 104.73(49.38) |
|  | Atorvastatin plus Torcetrapib | 450 | 46.8(12) | 47.6 | 138.4(35.5) | 52.9(12.7) | 213.0(39.3) | 100.33(46.11) |
| John J.P. Kastelein et. al,2015[14] | Anacetrapib100 mg | 204 | 55(11·8) | 59 | 127.61(30.94) | 54.14(15.47) | NA | 42.54(23.20) |
|  | Placebo | 102 | 55·7(11·9) | 49 | 131.48(46.40) | 54.14(15.47) | NA | 46.40(27.07) |
| A. Michael Lincoff et. al,2017[15] | Evacetrapib | 6038 | 64.8(9.4) | 77 | 81.6(28.4) | 45.3(11.7) | NA | 134.00(62.28) |
|  | Placebo | 6054 | 65.0(9.5) | 77 | 81.1(27.8) | 45.3(11.7) | NA | 133.33(62.28) |
| Thomas F. Lu ? scher et. al,2012[16] | Placebo | 234 | 61.9(7.92) | 90 | 79.31(17.67) | 38.48(7.15) | 147.02(21.77) | 63.96(28.35) |
|  | Dalcetrapib | 232 | 62.3(7.05) | 91 | 81.52(21.38) | 39.17(7.35) | 152.55(25.72) | 70.34(35.42) |
| Margaret E. Brousseau et. al,2004[17] | Atorvastatin plusTorcetrapib | 9 | 51(10) | 88.9 | 90(24) | 32(7) | 152(20) | 145(94) |
|  | Torcetrapib Alone | 10 | 49(13) | 90 | 134(25) | 33(6) | 203(32) | 162(89) |
| Christopher P. Cannon et. al,2010[18] | Anacetrapib | 811 | 62.5(8.7) | 77.6 | 81.4(21.3) | 40.5(9.3) | NA | NA |
|  | Placebo | 812 | 62.9(9.0) | 76.1 | 82.2(20.7) | 40.4(9.1) | NA | NA |
| Gregory G. Schwartz et. al,2012[19] | Placebo | 7933 | 60.1(9.1) | 81 | 75.8(25.9) | 42.2(11.5) | NA | 133.0(73.6) |
|  | Dalcetrapib | 7938 | 60.3(9.1) | 80 | 76.4(26.4) | 42.5(11.7) | NA | 134.2(73.6) |
| Stephen J. Nicholls et. al,2008[20] | Atorvastatin plus Placebo | 446 | 57.4(9.0) | 71.5 | 84.3(18.9) | 45.2(11.2) | 157.5(27.1) | 127.63(60.24) |
|  | Atorvastatin plus Torcetrapib | 464 | 56.8(9.1) | 71.3 | 83.1(19.7) | 46.0(12.8) | 157.8(27.6) | 129.83(67.30) |
| Stephen J. Nicholls et. al,2011[21] | Placebo | 38 | 55.2(10.5) | 47.4 | 147.3(21.6) | 53.0(11.8) | NA | 134.03(92.84) |
|  | Evacetrapib 30 mg/d | 40 | 58.5(11.1) | 42.5 | 143.5(26.0) | 54.7(12.0) | NA | 127.07(47.67) |
|  | Evacetrapib 100 mg/d | 38 | 58.5(9.2) | 42.1 | 148.0(25.0) | 57.0(14.1) | NA | 125.03(58.63) |
|  | Evacetrapib 500 mg/d | 40 | 58.8(12.2) | 47.5 | 135.7(26.0) | 54.7(16.3) | NA | 123.83(77.97) |
|  | Atorvastatin 20 mg/d with Placebo | 41 | 57.8(11.3) | 36.6 | 139.0(26.7) | 53.9(17.0) | NA | 134.63(75.52) |
|  | Atorvastatin 20 mg/d with Evacetrapib100 mg/d | 35 | 57.4(11.8) | 48.6 | 143.6(26.0) | 55.7(18.2) | NA | 122.80(66.40) |
|  | Simvastatin 40 mg/d with Placebo | 41 | 61.3(10.0) | 29.3 | 154.8(35.1) | 57.3(16.2) | NA | 127.83(62.61) |
|  | Simvastatin 40 mg/d with Evacetrapib100 mg/d | 40 | 58.4(9.0) | 37.5 | 143.7(29.1) | 53.7(13.6) | NA | 131.97(66.36) |
|  | Rosuvastatin 10 mg/d with Placebo | 39 | 57.4(12.6) | 66.7 | 141.6(23.8) | 53.5(15.2) | NA | 134.63(65.42) |
|  | Rosuvastatin 10 mg/d with Evacetrapib 100 mg/d | 41 | 59.7(10.1) | 43.9 | 145.7(21.8) | 57.8(18.1) | NA | 122.52(60.61) |
| Stephen J. Nicholls et. al, 2016[22] | Placebo | 38 | 55.2(10.5) | 47.4 | 147.3(21.6) | 53.0(11.8) | NA | 134(92.84) |
|  | Evacetrapib 30 mg | 40 | 58.5(11.1) | 42.5 | 143.5(26.0) | 54.7(12.0) | NA | 127.07(47.67) |
|  | Evacetrapib 100 mg | 38 | 58.5(9.2) | 42.1 | 148.0(25.0) | 57.0(14.1) | NA | 125.03(58.63) |
|  | Evacetrapib 500 mg | 40 | 58.8(12.2) | 47.5 | 135.7(26.0) | 54.7(16.3) | NA | 123.83(77.97) |
|  | Pooled Evacetrapib | 118 | 58.6(10.8) | 44.1 | 142.3(26.0) | 55.4(14.2) | NA | 125.00(55.84) |
|  | Pooled Statin | 121 | 58.9(11.4) | 43.8 | 145.2(29.6) | 54.9(16.1) | NA | 133.17(69.10) |
|  | Pooled Evacetrapib with statin | 116 | 58.5(10.2) | 43.1 | 144.4(25.6) | 55.8(16.7) | NA | 127.40(65.54) |
| Stephen J. Nicholls et. al, 2017[23] | Evacetrapib+Ezetimibe+Atorvastatin | 366 | 63.4(9.2) | 66.1 |  |  |  |  |
| Belinda Schludi et. al, 2022[24] | Placebo Q2W | 76 | 58.4(9.7) | 56.6 | 138.3(27.4) | 54.0(17.2) | NA | 140.7(73.4) |
|  | Placebo QM | 107 | 55.9(12.6) | 45.8 | 140.7(30.9: | 51.8(13.6) | NA | 136.6(74.4) |
|  | Evolocumab 140mg Q2W | 80 | 59.2(9.7) | 43.8 | 132.7(21.9) | 52.2(14.8) | NA | 134.9(54.7) |
|  | Evolocumab 420mg QM | 109 | 56.8(10.2) | 51.4 | 141.4(35.6) | 50.8(14.3) | NA | 141.7(67.4) |
| Christie M. Ballantyne et. al, 2023[25] | Placebo | 40 | 60.6(8.46) | 65 | 115.5(35.013) | 46(8.579) | NA | 159(71.881) |
|  | Obicetrapib 10mg | 26 | 64.8(7.24) | 65.4 | 106(38.874) | 58.25(20.952) | NA | 151(58.564) |
|  | Obicetrapib 10mg + Ezetimibe 10mg | 31 | 63.5(9.08) | 61.3 | 97(21.911) | 49(11.686) | NA | 147.750(58.186) |
| Gisette Reyes-Soffer et. al, 2016[26] | Atorvastatin-Anacetrapib | 29 | 46.7(10.1) | 69 | 139.7(26.9) | 50.5(13.9) | 215.8(31.6) | 114.33(45.22) |
|  | Placebo-Anacetrapib | 10 | 50.6(11.3) | 60 | 130.9(20.5) | 45.6(12.5) | 207.0(27.0) | 135(73.10) |
|  | Dalcetrapib | 969 | 63(10) | 79 | 80.9(30.9) | 42.5(12.0) | NA | 141(73) |
|  | Placebo | 3170 | 60.(9) | 81 | 75.3(24.7) | 42.2(11.2) | NA | 131(69) |
| Evan A. Stein et. al, 2010[28] | Placebo (core phase) | 46 | 60.2(7.5) | 83 | 76.9(20.7) | 41.0(11.4) | 144.2(26.03) | 131.7(69.72) |
|  | Dalcetrapib 900mg (core phase) | 89 | 61.2(7.76) | 76 | 76.9(16.4) | 41.4(9.31) | 147.3(22.26) | 150.2(92.57) |
|  | Placebo (extension phase) | 25 | 60.8(7.83) | 88 | 78.9(23.7) | 41.8(12.5) | 150.6(29.25) | 149.9(80.46) |
|  | Dalcetrapib 900mg (extension phase) | 52 | 60.6(7.03) | 77 | 74.2(17.1) | 42.4(9.42) | 143.8(19.86) | 136.0(56.48) |
| Jean-Claude Tardif et. al, 2014[29] | Dalcetrapib clinical event | 390 | 61.8(9.3) | 81 | 79.61(27.73) | 43.08(11.75) | 151.23(34.92) | 143.11(80.19) |
|  | Dalcetrapib no event | 2455 | 60.3(9.0) | 79.1 | 76.09(24.98) | 43.20(11.67) | 145.51(31.21) | 131.60(71.64) |
|  | Placebo clinical event | 398 | 61.5(9.3) | 81.7 | 80.85(28.38) | 42.79(12.13) | 151.04(36.74) | 138.5(87.19) |
|  | Placebo no event | 2506 | 60.4(8.9) | 80.5 | 75.2(24.86) | 42.92(11.74) | 144.22(32.02) | 131.45(75.48) |
| Jean-Claude Tardif et. al, 2016[30] | Dalcetrapib | 2665 | 60.5(9.0) | 79.2 | 76.6(25.5) | 43.3(11.7) | 146.4(32) | 133.1(73.3) |
|  | Placebo | 2699 | 60.5(9.0) | 81.4 | 76.1(25.6) | 43(11.7) | 145.4(33.1) | 133.1(78.4) |
| Tamio Teramoto et. al, 2013[31] | Placebo | 40 | 50.5(11.8) | 75 | 153.8(16.3) | 49.9(5.8) | 231.9(21.1) | N/A |
|  | Anacetrapib 10mg | 41 | 56.3(11) | 61 | 155.8(15.9) | 50.6(6.7) | 235.5(16) | N/A |
|  | Anacetrapib 40mg | 40 | 51.3(10.1) | 72.5 | 154.4(16.7) | 50.7(6.7) | 234.2(20.5) | N/A |
|  | Anacetrapib 100mg | 40 | 55(10.4) | 70 | 153(14.3) | 49.8(6) | 231.1(19.7) | N/A |
|  | Anacetrapib 300mg | 42 | 56.3(10.2) | 64.3 | 159.3(17.4) | 50.6(6.4) | 237.5(24.1) | N/A |
|  | Atorvastatin 10mg | 40 | 54.7(12.8) | 65 | 158.6(21.5) | 49.6(6.6) | 237.7(24) | N/A |
|  | Anacetrapib 10mg+Atorvastatin 10mg | 40 | 52.8(9.5) | 60 | 157.1(14.5) | 50.3(6.4) | 237.4(25) | N/A |
|  | Anacetrapib 40mg+Atorvastatin 10mg | 41 | 53.6(11.5) | 63.4 | 158(20.2) | 49.8(6.6) | 238.5(19.7) | N/A |
|  | Anacetrapib 100mg+Atorvastatin 10mg | 42 | 53(9.8) | 64.3 | 156.6(16.6) | 49.5(7.9) | 237.5(19.7) | N/A |
|  | Anacetrapib 300mg+Atorvastatin 10mg | 41 | 55.2(10.2) | 80.5 | 153.8(16) | 49.3(6.2) | 233.5(21) | N/A |
| Tamio Teramoto et. al, 2014[32] | Placebo | 28 | 50(9.6) | 64 | 140(27) | 51(14) | NA | 147(63.627) |
|  | Evacetrapib 30mg | 27 | 49(11) | 70 | 144(24) | 50(13) | NA | 178.5(82.642) |
|  | Evacetrapib 100mg | 28 | 48(12) | 64 | 144(23) | 52(16) | NA | 163.750(75.308) |
|  | Evacetrapib 500mg | 27 | 49(8.1) | 67 | 143(30) | 49(11) | NA | 175.500(81.139) |
|  | Atorvastatin 10mg | 27 | 49(8.8) | 70 | 134(32) | 49(14) | NA | 166.25(68.367) |
|  | Atorvastation 10mg + Evacetrapib 100mg | 28 | 50(10) | 64 | 140(20) | 52(14) | NA | 145.5(61.141) |
| Tamio Teramoto et. al, 2017[33] | Evacetrapib 130mg | 27 | 52.2(10.2) | 74.1 | 158.1(21.5) | 53.0(10.3) | NA | 131.6(61.9) |
|  | Placebo | 27 | 53.3(10.1) | 63 | 162.3(23.3) | 58.8(11.9) | NA | 125.0(68.2) |

**Supplementary Table 1: Population Baseline Characteristics of Included Studies with References**

**References:**

1.Harada-Shiba, M., Davdison, M. H., Ditmarsch, M., Hsieh, A., Wuerdeman, E., Kling, D., Nield, A., Dicklin, M. R., Nakata, A., Sueyoshi, A., Kuroyanagi, S., & Kastelein, J. J. P. (2024). Obicetrapib as an Adjunct to Stable Statin Therapy in Japanese Subjects: Results from a Randomized Phase 2 Trial. *Journal* of atherosclerosis *and*

*thrombosis*, *31*(10), 1386-1397.

<https://doi.org/10.5551/jat.64828>

*2.* Millar, J. S., Reyes-Soffer, G., Jumes, P., Dunbar, R. L., deGoma, E. M., Baer, A. L., Karmally, W., Donovan, D. S., Rafeek, H., Pollan, L., Tohyama, J., Johnson-Levonas, A. O., Wagner, J. A., Holleran, S., Obunike, J., Liu, Y., Ramakrishnan, R., Lassman, M. E., Gutstein, D. E., Ginsberg, H. N., ... Rader, D. J. (2015). Anacetrapib lowers LDL by increasing ApoB clearance in mildly hypercholesterolemic subjects. The *Journal* of clinical *investigation, 125*(6), 2510-2522.

<https://doi.org/10.1172/JCI80025>

3. Arsenault, B. J., Petrides, F., Tabet, F., Bao, W., Hovingh, G. K., Boekholdt, S. M., Ramin- Mangata, S., Meilhac, O., DeMicco, D., Rye, K. A., Waters, D. D., Kastelein, J. J. P., Barter, P., & Lambert, G. (2018). Effect of atorvastatin, cholesterol ester transfer protein inhibition, and diabetes mellitus on circulating proprotein subtilisin kexin type 9 and lipoprotein(a) levels in patients at high cardiovascular risk. *Journal* of clinical lipidology*, 12*(1), 130–136. <https://doi.org/10.1016/j.jacl.2017.10.001>

4. Barter, P. J., Caulfield, M., Eriksson, M., Grundy, S. M., Kastelein, J. J., Komajda, M., Lopez- Sendon, J., Mosca, L., Tardif, J. C., Waters, D. D., Shear, C. L., Revkin, J. H., Buhr, K. A., Fisher, M. R., Tall, A. R., Brewer, B., & ILLUMINATE Investigators (2007). Effects of torcetrapib in patients at high risk for coronary events. The New England journal of medicine, 357(21), 2109-2122.

<https://doi.org/10.1056/NEJMoa0706628>

5.Bloomfield, D., Carlson, G. L., Sapre, A., Tribble, D., McKenney, J. M., Littlejohn, T. W., 3rd, Sisk, C. M., Mitchel, Y., & Pasternak, R. C. (2009). Efficacy and safety of the cholesteryl ester transfer protein inhibitor anacetrapib as monotherapy and coadministered with atorvastatin in dyslipidemic patients. American heart *journal,* 157(2), 352–360.e2.

<https://doi.org/10.1016/j.ahj.2008.09.022>

6. Brinton, E. A., Kher, U., Shah, S., Cannon, C. P., Davidson, M., Gotto, A. M., Ashraf, T. B., McCrary Sisk, C., Dansky, H., Mitchel, Y., Barter, P., & DEFINE Investigators (2015). Effects of anacetrapib on plasma lipids in specific patient subgroups in the DEFINE (Determining the Efficacy and Tolerability of CETP INhibition with AnacEtrapib) trial. *Journal* of *clinical lipidology,* 9(1), 65–71.

<https://doi.org/10.1016/j.jacl.2014.10.005>

7.Cannon, C. P., Shah, S., Dansky, H. M., Davidson, M., Brinton, E. A., Gotto, A. M., Stepanavage, M., Liu, S. X., Gibbons, P., Ashraf, T. B., Zafarino, J., Mitchel, Y., Barter, P., & Determining the Efficacy and Tolerability Investigators (2010). Safety of anacetrapib in patients with or at high risk for coronary heart disease. *The* New England *journal* of *medicine,* 363(25), 2406-2415.

<https://doi.org/10.1056/NEJMoa1009744>

8. Davidson, M. H., McKenney, J. M., Shear, C. L., & Revkin, J. H. (2006). Efficacy and safety of torcetrapib, a novel cholesteryl ester transfer protein inhibitor, in individuals with below- average high-density lipoprotein cholesterol levels. *Journal* of the American College *of Cardiology, 48*(9), 1774-1781.

<https://doi.org/10.1016/j.jacc.2006.06.067>

9. Schwartz, G. G., Leiter, L. A., Ballantyne, C. M., Barter, P. J., Black, D. M., Kallend, D., Laghrissi-Thode, F., Leitersdorf, E., McMurray, J. J. V., Nicholls, S. J., Olsson, A. G., Preiss, D., Shah, P. K., Tardif, J. C., & Kittelson, J. (2020). Dalcetrapib Reduces Risk of New-Onset Diabetes in Patients With Coronary Heart Disease. *Diabetes* care, *43*(5), 1077-1084. <https://doi.org/10.2337/dc19-2204>

10. Gotto, A. M., Jr, Cannon, C. P., Li, X. S., Vaidya, S., Kher, U., Brinton, E. A., Davidson, M., Moon, J. E., Shah, S., Dansky, H. M., Mitchel, Y., Barter, P., & DEFINE Investigators (2014). Evaluation of lipids, drug concentration, and safety parameters following cessation of treatment with the cholesteryl ester transfer protein inhibitor anacetrapib in patients with or at high risk for coronary heart disease. *The* American *journal* of *cardiology,* 113(1), 76–83. <https://doi.org/10.1016/j.amjcard.2013.08.041>

11.HPS3/TIMI55–REVEAL Collaborative Group, Bowman, L., Hopewell, J. C., Chen, F., Wallendszus, K., Stevens, W., Collins, R., Wiviott, S. D., Cannon, C. P., Braunwald, E., Sammons, E., & Landray, M. J. (2017). Effects of Anacetrapib in Patients with Atherosclerotic Vascular Disease. *The New England journal of medicine*, *377*(13), 1217–1227. <https://doi.org/10.1056/NEJMoa1706444>

12.Hovingh, G. K., Kastelein, J. J., van Deventer, S. J., Round, P., Ford, J., Saleheen, D., Rader, D. J., Brewer, H. B., & Barter, P. J. (2015). Cholesterol ester transfer protein inhibition by TA-8995 in patients with mild dyslipidaemia (TULIP): a randomised, double-blind, placebo-controlled phase 2 trial. *Lancet (London, England)*, *386*(9992), 452–460. <https://doi.org/10.1016/S0140-6736(15)60158-1>

13.Kastelein, J. J., van Leuven, S. I., Burgess, L., Evans, G. W., Kuivenhoven, J. A., Barter, P. J., Revkin, J. H., Grobbee, D. E., Riley, W. A., Shear, C. L., Duggan, W. T., Bots, M. L., & RADIANCE 1 Investigators (2007). Effect of torcetrapib on carotid atherosclerosis in familial hypercholesterolemia. *The New England journal of medicine*, *356*(16), 1620–1630. <https://doi.org/10.1056/NEJMoa071359>

14.Kastelein, J. J., Besseling, J., Shah, S., Bergeron, J., Langslet, G., Hovingh, G. K., Al-Saady, N., Koeijvoets, M., Hunter, J., Johnson-Levonas, A. O., Fable, J., Sapre, A., & Mitchel, Y. (2015). Anacetrapib as lipid-modifying therapy in patients with heterozygous familial hypercholesterolaemia (REALIZE): a randomised, double-blind, placebo-controlled, phase 3 study. *Lancet (London, England)*, *385*(9983), 2153–2161. <https://doi.org/10.1016/S0140-6736(14)62115-2>

15.Lincoff, A. M., Nicholls, S. J., Riesmeyer, J. S., Barter, P. J., Brewer, H. B., Fox, K. A. A., Gibson, C. M., Granger, C., Menon, V., Montalescot, G., Rader, D., Tall, A. R., McErlean, E., Wolski, K., Ruotolo, G., Vangerow, B., Weerakkody, G., Goodman, S. G., Conde, D., McGuire, D. K., … ACCELERATE Investigators (2017). Evacetrapib and Cardiovascular Outcomes in High-Risk Vascular Disease. *The New England journal of medicine*, *376*(20), 1933–1942. <https://doi.org/10.1056/NEJMoa1609581>

16.Lüscher, T. F., Taddei, S., Kaski, J. C., Jukema, J. W., Kallend, D., Münzel, T., Kastelein, J. J., Deanfield, J. E., & dal-VESSEL Investigators (2012). Vascular effects and safety of dalcetrapib in patients with or at risk of coronary heart disease: the dal-VESSEL randomized clinical trial. *European heart journal*, *33*(7), 857–865. <https://doi.org/10.1093/eurheartj/ehs019>

17.Brousseau, M. E., Schaefer, E. J., Wolfe, M. L., Bloedon, L. T., Digenio, A. G., Clark, R. W., Mancuso, J. P., & Rader, D. J. (2004). Effects of an inhibitor of cholesteryl ester transfer protein on HDL cholesterol. *The New England journal of medicine*, *350*(15), 1505–1515. <https://doi.org/10.1056/NEJMoa031766>

18.Cannon, C. P., Shah, S., Dansky, H. M., Davidson, M., Brinton, E. A., Gotto, A. M., Stepanavage, M., Liu, S. X., Gibbons, P., Ashraf, T. B., Zafarino, J., Mitchel, Y., Barter, P., & Determining the Efficacy and Tolerability Investigators (2010). Safety of anacetrapib in patients with or at high risk for coronary heart disease. *The New England journal of medicine*, *363*(25), 2406–2415. <https://doi.org/10.1056/NEJMoa1009744>

19.Schwartz, G. G., Olsson, A. G., Abt, M., Ballantyne, C. M., Barter, P. J., Brumm, J., Chaitman, B. R., Holme, I. M., Kallend, D., Leiter, L. A., Leitersdorf, E., McMurray, J. J., Mundl, H., Nicholls, S. J., Shah, P. K., Tardif, J. C., Wright, R. S., & dal-OUTCOMES Investigators (2012). Effects of dalcetrapib in patients with a recent acute coronary syndrome. *The New England journal of medicine*, *367*(22), 2089–2099. <https://doi.org/10.1056/NEJMoa1206797>

20.Nicholls, S. J., Tuzcu, E. M., Brennan, D. M., Tardif, J. C., & Nissen, S. E. (2008). Cholesteryl ester transfer protein inhibition, high-density lipoprotein raising, and progression of coronary atherosclerosis: insights from ILLUSTRATE (Investigation of Lipid Level Management Using Coronary Ultrasound to Assess Reduction of Atherosclerosis by CETP Inhibition and HDL Elevation). *Circulation*, *118*(24), 2506–2514. <https://doi.org/10.1161/CIRCULATIONAHA.108.790733>

21.Nicholls, S. J., Brewer, H. B., Kastelein, J. J., Krueger, K. A., Wang, M. D., Shao, M., Hu, B., McErlean, E., & Nissen, S. E. (2011). Effects of the CETP inhibitor evacetrapib administered as monotherapy or in combination with statins on HDL and LDL cholesterol: a randomized controlled trial. *JAMA*, *306*(19), 2099–2109. <https://doi.org/10.1001/jama.2011.1649>

22. Nicholls, S. J., Ruotolo, G., Brewer, H. B., Wang, M. D., Liu, L., Willey, M. B., Deeg, M. A., Krueger, K. A., & Nissen, S. E. (2016). Evacetrapib alone or in combination with statins lowers lipoprotein(a) and total and small LDL particle concentrations in mildly hypercholesterolemic patients. *Journal of clinical lipidology*, *10*(3), 519–527.e4. <https://doi.org/10.1016/j.jacl.2015.11.014>

23. Nicholls, S. J., Ray, K. K., Ballantyne, C. M., Beacham, L. A., Miller, D. L., Ruotolo, G., Nissen, S. E., Riesmeyer, J. S., & ACCENTUATE Investigators (2017). Comparative effects of cholesteryl ester transfer protein inhibition, statin or ezetimibe on lipid factors: The ACCENTUATE trial. *Atherosclerosis*, *261*, 12–18. <https://doi.org/10.1016/j.atherosclerosis.2017.04.008>

24. Schludi, B., Giugliano, R. P., Sabatine, M. S., Raal, F. J., Teramoto, T., Koren, M. J., Stein, E. A., Wang, H., & Monsalvo, M. L. (2022). Time-averaged low-density lipoprotein cholesterol lowering with evolocumab: Pooled analysis of phase 2 trials. *Journal of clinical lipidology*, *16*(4), 538–543. <https://doi.org/10.1016/j.jacl.2022.05.069>

25. Ballantyne, C. M., Ditmarsch, M., Kastelein, J. J., Nelson, A. J., Kling, D., Hsieh, A., Curcio, D. L., Maki, K. C., Davidson, M. H., & Nicholls, S. J. (2023). Obicetrapib plus ezetimibe as an adjunct to high-intensity statin therapy: A randomized phase 2 trial. *Journal of clinical lipidology*, *17*(4), 491–503. <https://doi.org/10.1016/j.jacl.2023.05.098>

26. Reyes-Soffer, G., Millar, J. S., Ngai, C., Jumes, P., Coromilas, E., Asztalos, B., Johnson-Levonas, A. O., Wagner, J. A., Donovan, D. S., Karmally, W., Ramakrishnan, R., Holleran, S., Thomas, T., Dunbar, R. L., deGoma, E. M., Rafeek, H., Baer, A. L., Liu, Y., Lassman, M. E., Gutstein, D. E., … Ginsberg, H. N. (2016). Cholesteryl Ester Transfer Protein Inhibition With Anacetrapib Decreases Fractional Clearance Rates of High-Density Lipoprotein Apolipoprotein A-I and Plasma Cholesteryl Ester Transfer Protein. *Arteriosclerosis, thrombosis, and vascular biology*, *36*(5), 994–1002. <https://doi.org/10.1161/ATVBAHA.115.306680>

27. Schwartz, G. G., Ballantyne, C. M., Barter, P. J., Kallend, D., Leiter, L. A., Leitersdorf, E., McMurray, J. J. V., Nicholls, S. J., Olsson, A. G., Shah, P. K., Tardif, J. C., & Kittelson, J. (2018). Association of Lipoprotein(a) With Risk of Recurrent Ischemic Events Following Acute Coronary Syndrome: Analysis of the dal-Outcomes Randomized Clinical Trial. *JAMA cardiology*, *3*(2), 164–168. <https://doi.org/10.1001/jamacardio.2017.3833>

28. Stein, E. A., Roth, E. M., Rhyne, J. M., Burgess, T., Kallend, D., & Robinson, J. G. (2010). Safety and tolerability of dalcetrapib (RO4607381/JTT-705): results from a 48-week trial. *European heart journal*, *31*(4), 480–488. <https://doi.org/10.1093/eurheartj/ehp601>

29. Tardif, J. C., Rhéaume, E., Lemieux Perreault, L. P., Grégoire, J. C., Feroz Zada, Y., Asselin, G., Provost, S., Barhdadi, A., Rhainds, D., L'Allier, P. L., Ibrahim, R., Upmanyu, R., Niesor, E. J., Benghozi, R., Suchankova, G., Laghrissi-Thode, F., Guertin, M. C., Olsson, A. G., Mongrain, I., Schwartz, G. G., … Dubé, M. P. (2015). Pharmacogenomic determinants of the cardiovascular effects of dalcetrapib. *Circulation. Cardiovascular genetics*, *8*(2), 372–382. <https://doi.org/10.1161/CIRCGENETICS.114.000663>

30. Tardif, J. C., Rhainds, D., Brodeur, M., Feroz Zada, Y., Fouodjio, R., Provost, S., Boulé, M., Alem, S., Grégoire, J. C., L'Allier, P. L., Ibrahim, R., Guertin, M. C., Mongrain, I., Olsson, A. G., Schwartz, G. G., Rhéaume, E., & Dubé, M. P. (2016). Genotype-Dependent Effects of Dalcetrapib on Cholesterol Efflux and Inflammation: Concordance With Clinical Outcomes. *Circulation. Cardiovascular genetics*, *9*(4), 340–348. <https://doi.org/10.1161/CIRCGENETICS.116.001405>

31. Teramoto, T., Shirakawa, M., Kikuchi, M., Nakagomi, M., Tamura, S., Surks, H. K., McCrary Sisk, C., & Numaguchi, H. (2013). Efficacy and safety of the cholesteryl ester transfer protein inhibitor anacetrapib in Japanese patients with dyslipidemia. *Atherosclerosis*, *230*(1), 52–60. <https://doi.org/10.1016/j.atherosclerosis.2013.05.012>

32. Teramoto, T., Takeuchi, M., Morisaki, Y., Ruotolo, G., & Krueger, K. A. (2014). Efficacy, safety, tolerability, and pharmacokinetic profile of evacetrapib administered as monotherapy or in combination with atorvastatin in Japanese patients with dyslipidemia. *The American journal of cardiology*, *113*(12), 2021–2029. <https://doi.org/10.1016/j.amjcard.2014.03.045>

33. Teramoto, T., Kiyosue, A., Iimura, T., Takita, Y., Riesmeyer, J. S., & Murakami, M. (2017). Efficacy and Safety of Cholesteryl Ester Transfer Protein Inhibitor Evacetrapib Administered as Monotherapy in Japanese Patients With Primary Hypercholesterolemia. *Circulation journal : official journal of the Japanese Circulation Society*, *81*(11), 1686–1692. <https://doi.org/10.1253/circj.CJ-16-1325>
