# Supplementary material for: Comparative Effectiveness of Cholesteryl Ester Transfer Protein (CETP) Inhibitors on Lipid Profiles in Adults With Hyperlipidemia: A Comprehensive Systematic Review and Frequentist Network Meta‐Analysis of Randomized Controlled Trials
Source: Clin Cardiol. 2025 Sep 14;48(9):e70204. doi: 10.1002/clc.70204 (PMC12434180; doi:10.1002/clc.70204)
Supplement: Supplementary file 2 — Figure S1: Plot showing the risk of bias assessment (ROB2) of included studies. Figure S2: Funnel plot with Egger test showing the publication bias of included studies. Figure S3: Heatmap showing the heterogeneity and inconsistency results of interventions of included studies for the outcome LDL‐C. Figure S4: Heatmap showing the heterogeneity and inconsistency results of interventions of included studies for the outcome HDL‐C. Figure S5: Heatmap showing the heterogeneity and inconsistency results of interventions of included studies for the outcome TG. Figure S6: Heatmap showing the heterogeneity and inconsistency results of interventions of included studies for the outcome TC. Figure S7: Splitted forest plot showing differences among direct and indirect comparisons of intervention for the outcome LDL‐C. Figure S8: Splitted forest plot showing differences among direct and indirect comparisons of intervention for the outcome HDL‐C. Figure S9: Splitted forest plot showing differences among direct and indirect comparisons of intervention for the outcome TG. Figure S10: Splitted forest plot showing differences among direct and indirect comparisons of intervention for the outcome TC. [file CLC-48-e70204-s002.docx]

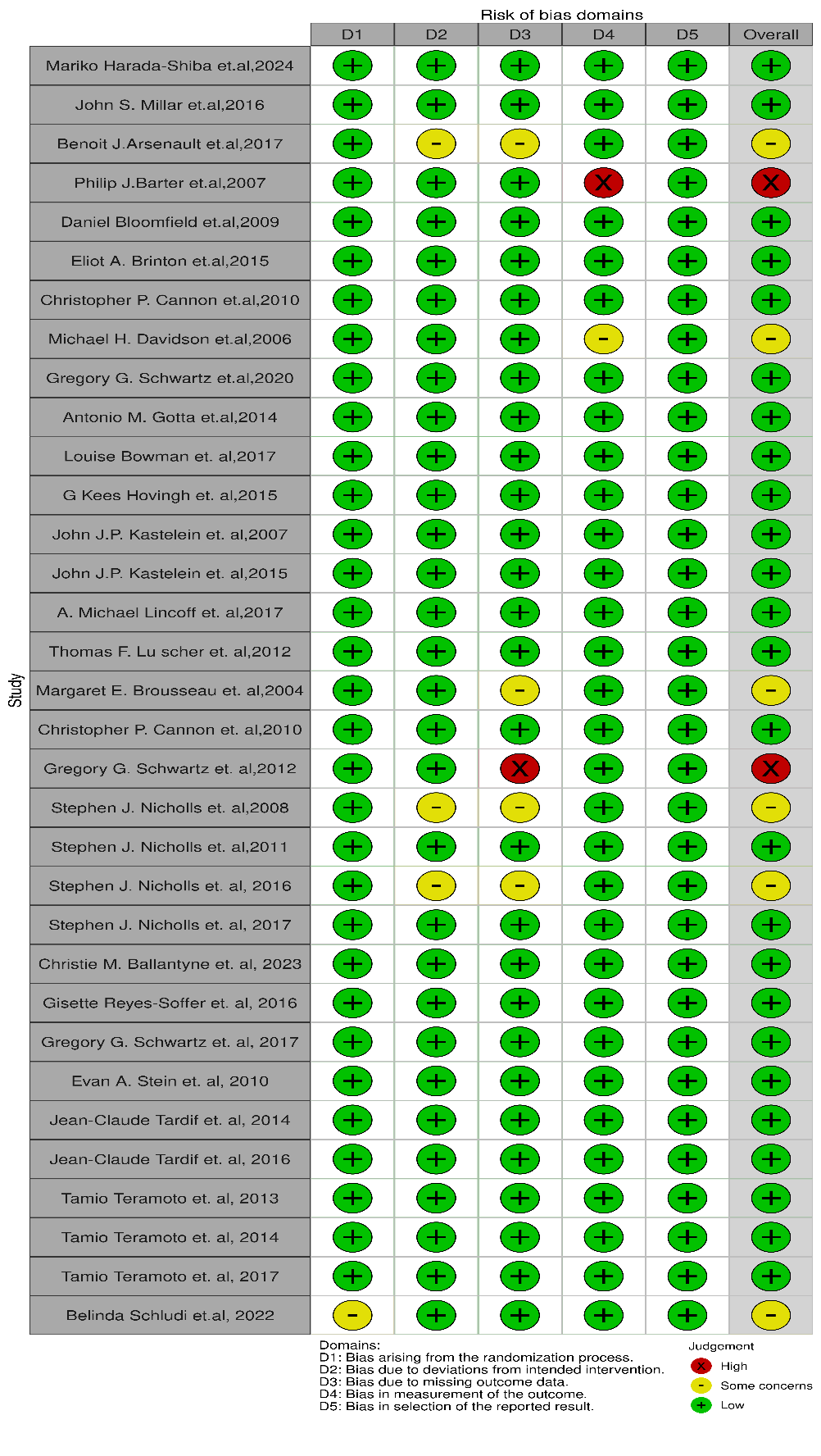

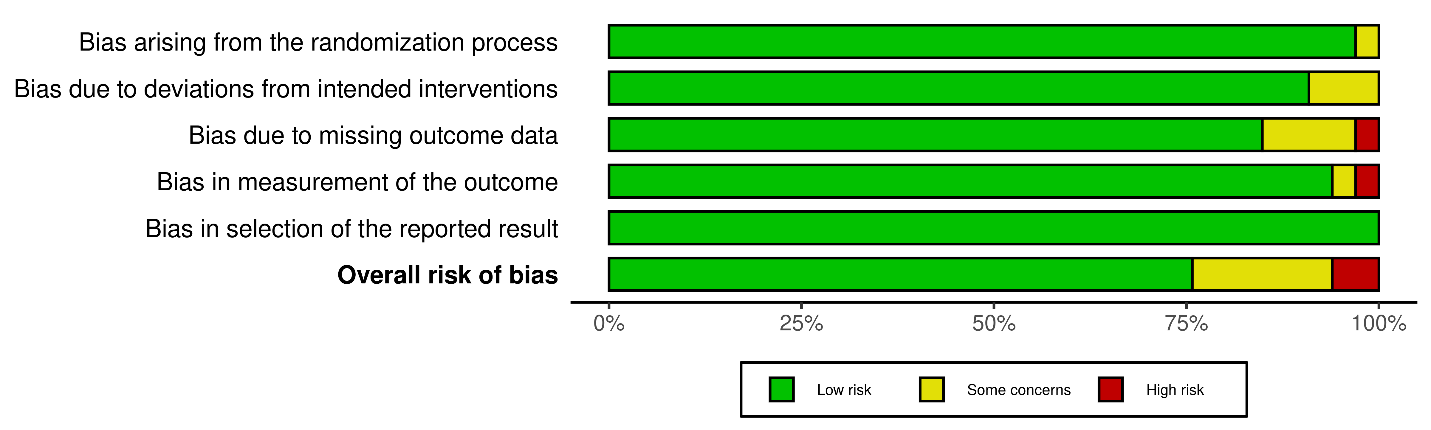


Supplementary Figure 1: Plot showing the risk of bias assessment (ROB2) of included studies.


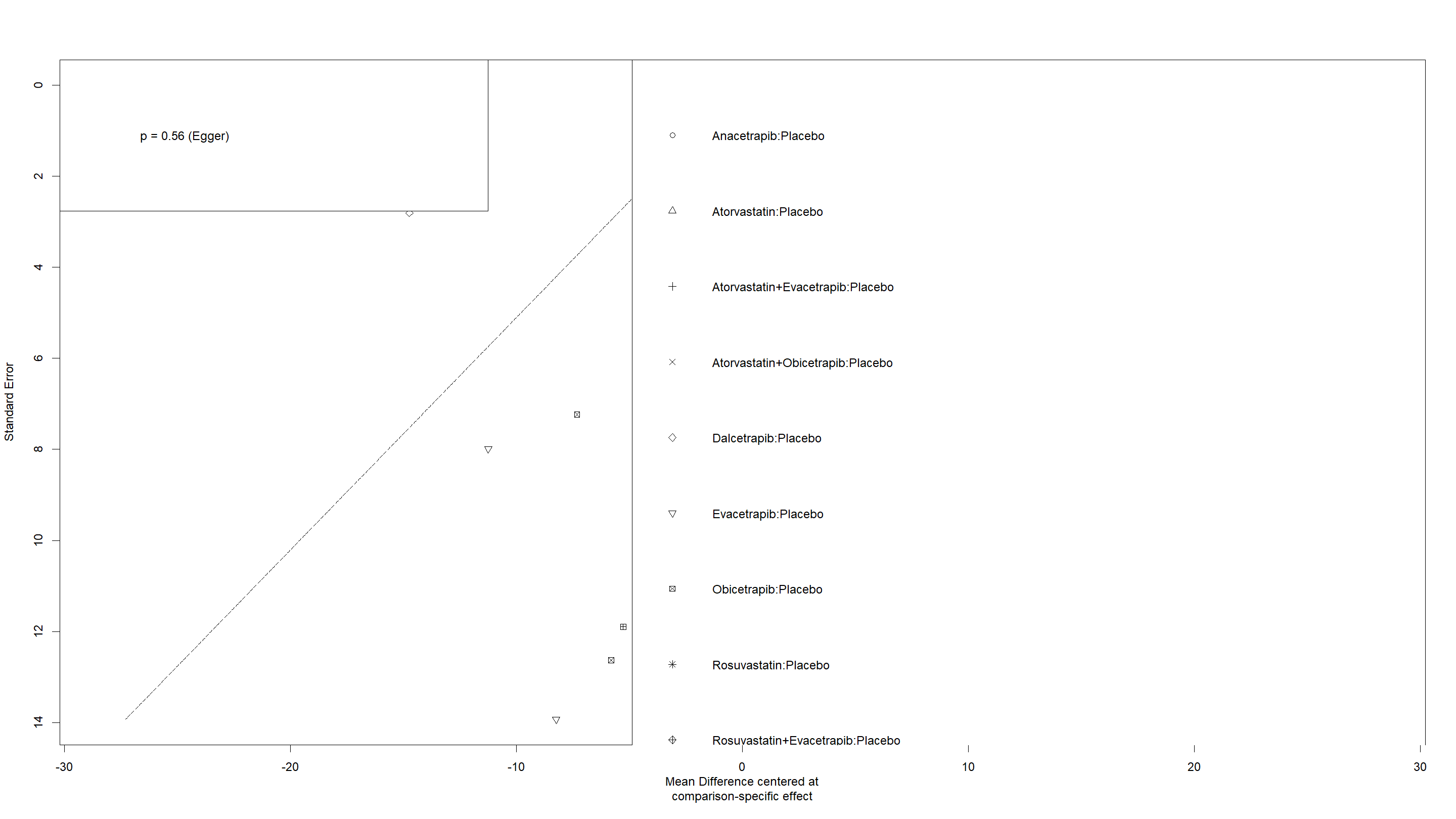


Supplementary Figure 2: Funnel plot with Egger test showing the publication bias of included studies.


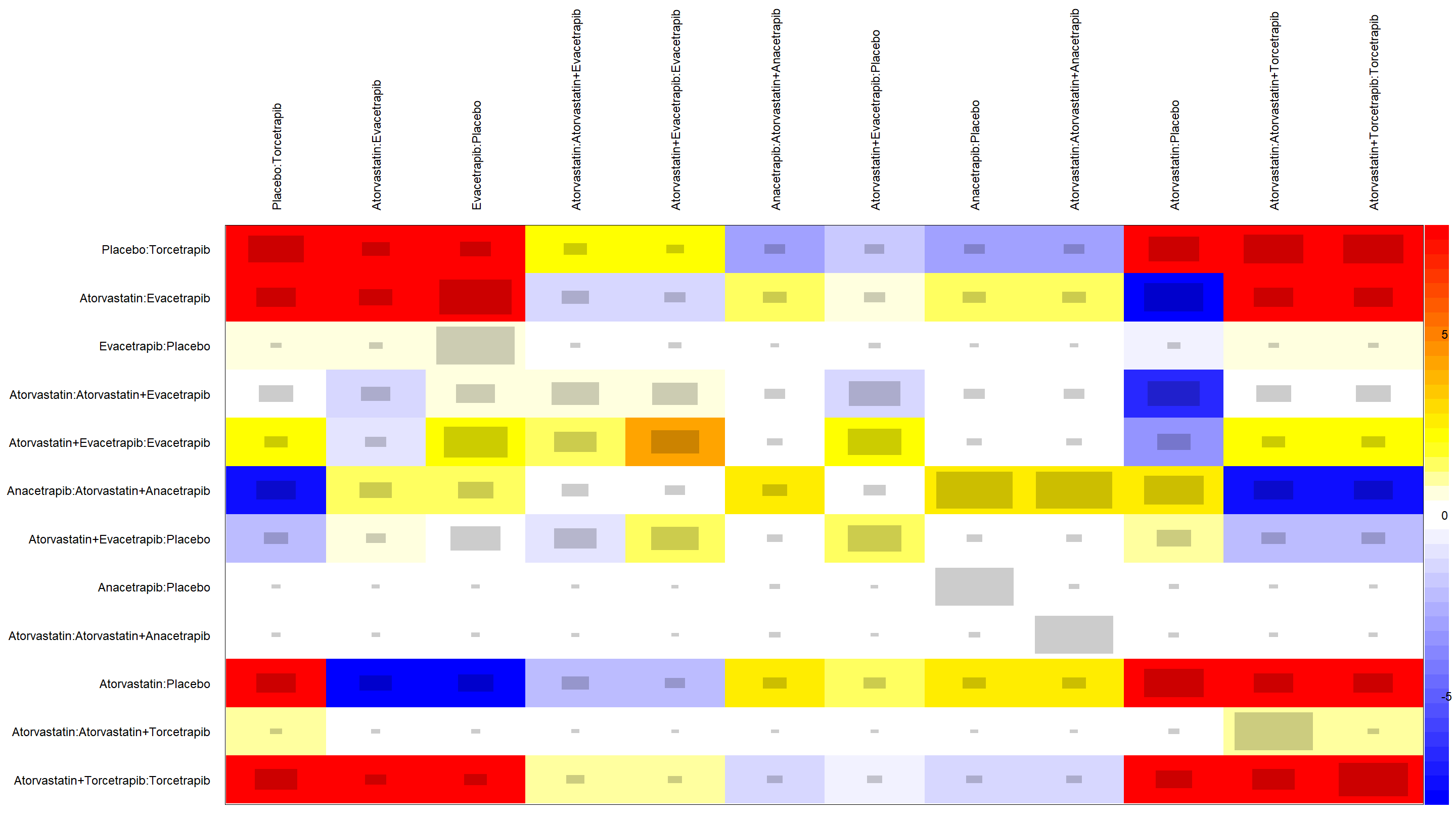


Supplementary Figure 3: Heatmap showing the heterogeneity and inconsistency results of interventions of included studies for the outcome LDL-C.


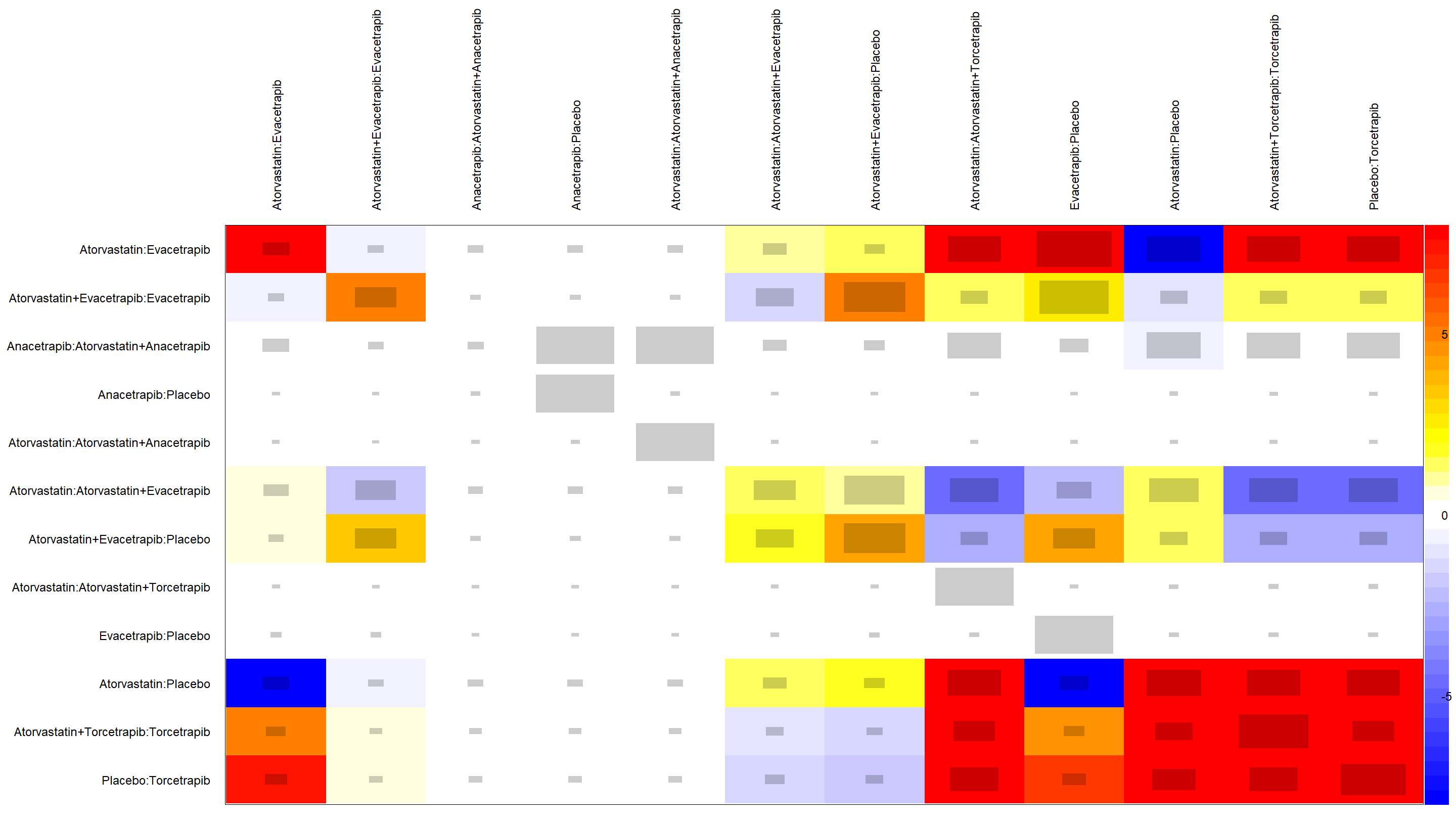


Supplementary Figure 4: Heatmap showing the heterogeneity and inconsistency results of interventions of included studies for the outcome HDL-C.


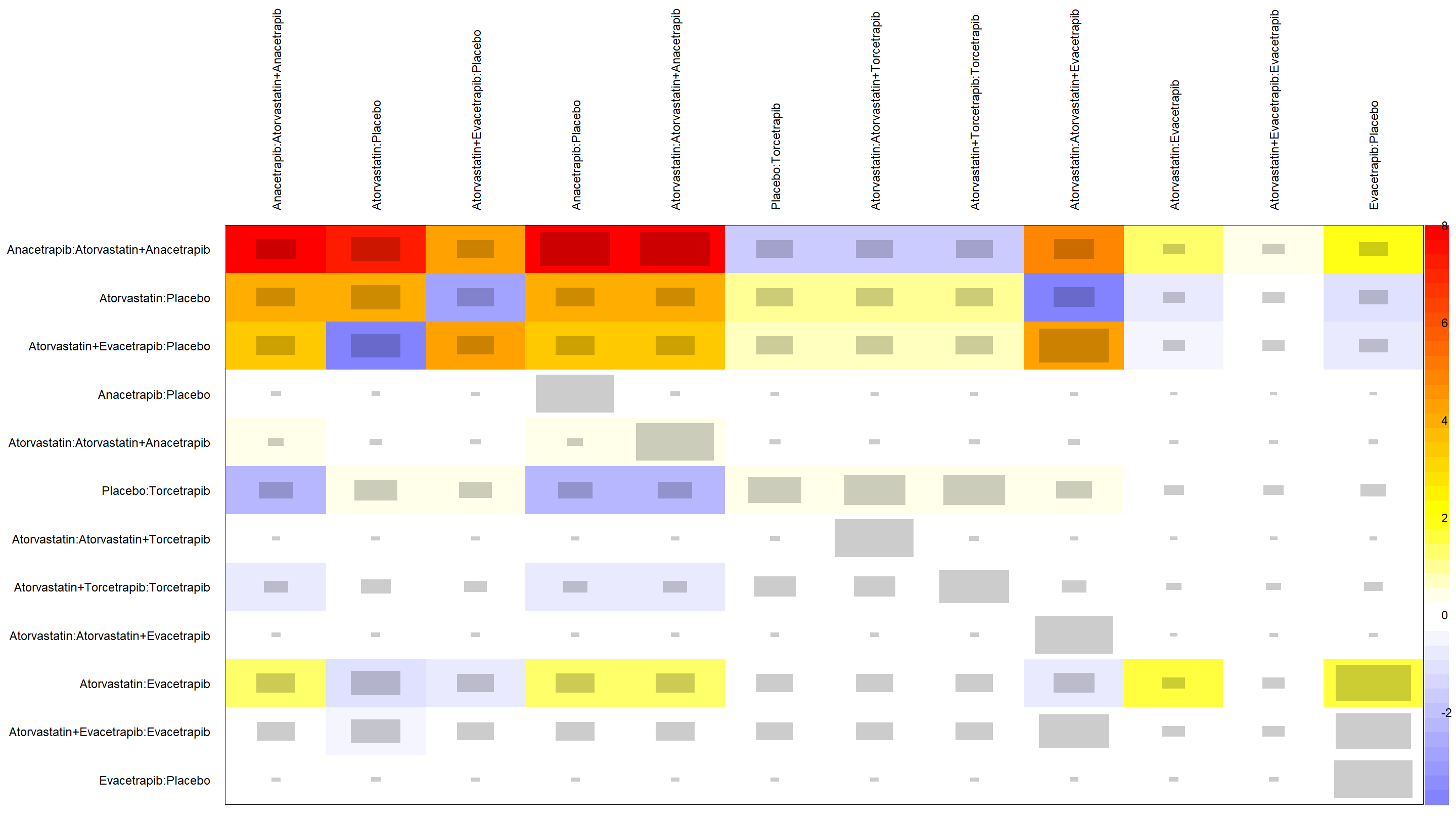


Supplementary Figure 5: Heatmap showing the heterogeneity and inconsistency results of interventions of included studies for the outcome TG.


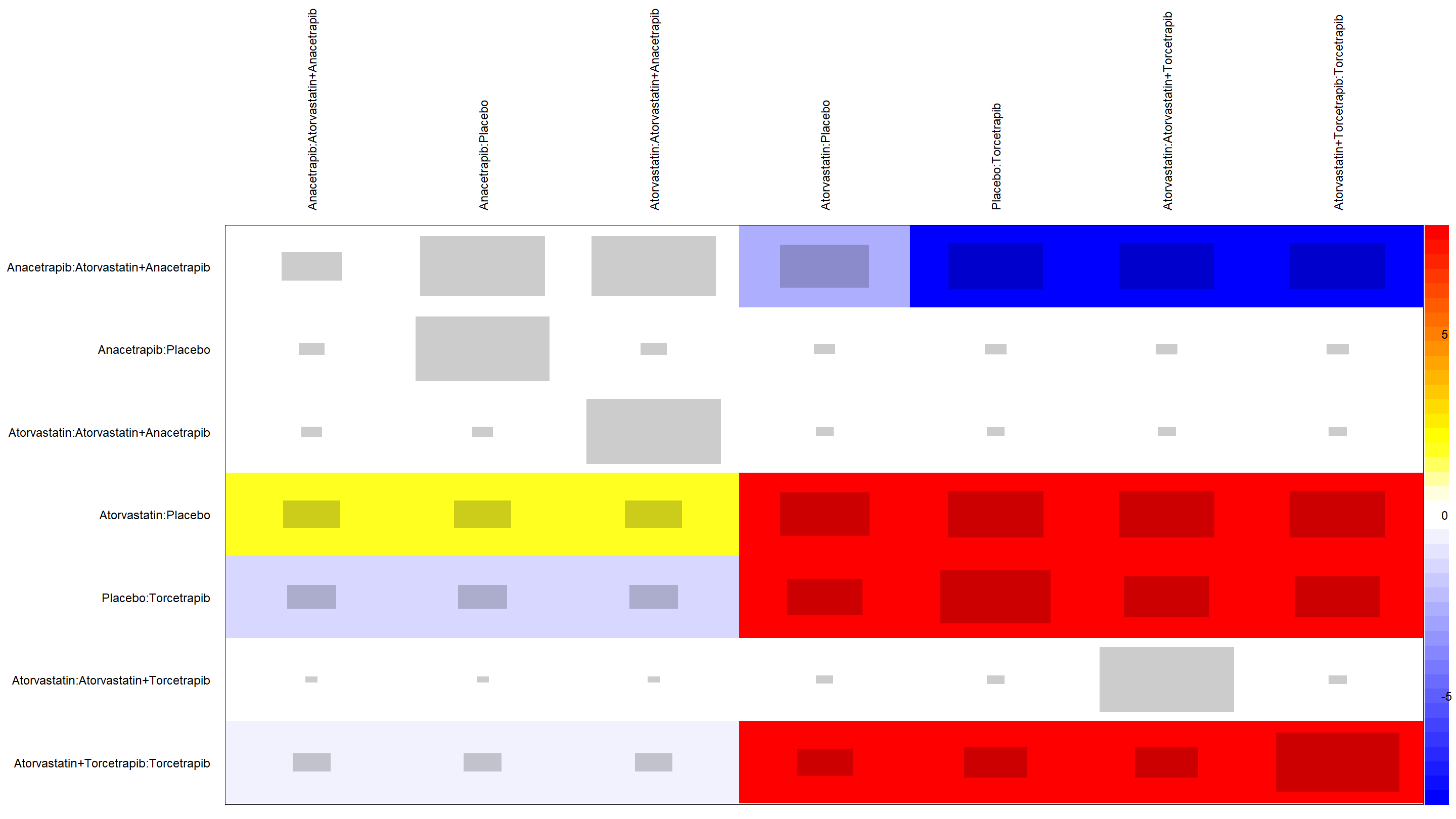


Supplementary Figure 6: Heatmap showing the heterogeneity and inconsistency results of interventions of included studies for the outcome TC.


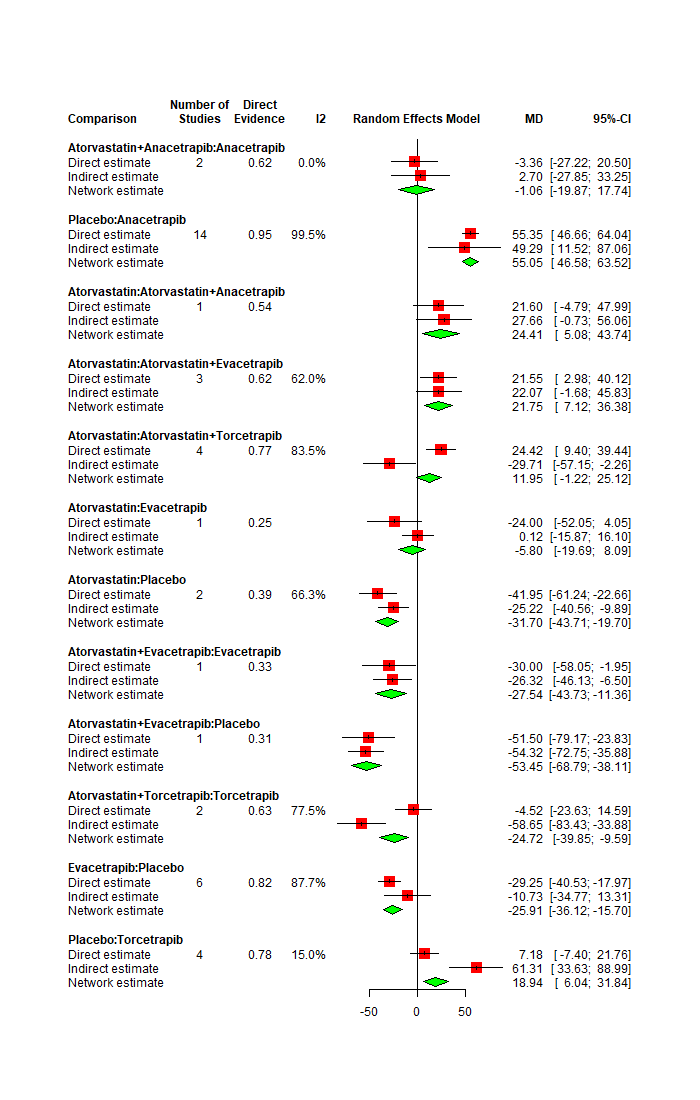


Supplementary Figure 7: Splitted forest plot showing differences among direct and indirect comparisons of intervention for the outcome LDL-C.


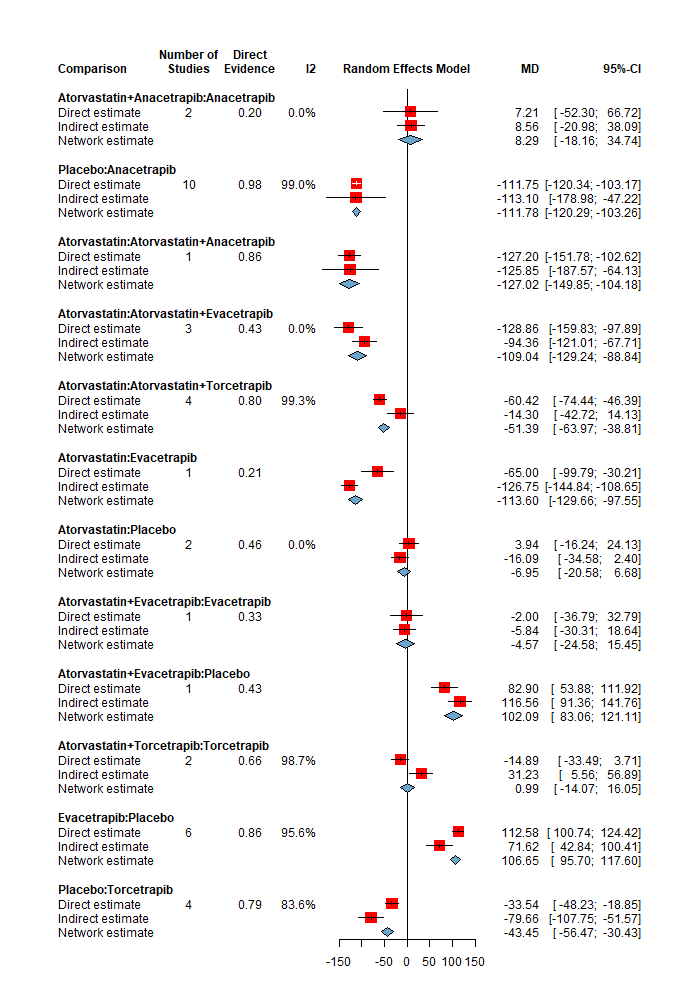


Supplementary Figure 8: Splitted forest plot showing differences among direct and indirect comparisons of intervention for the outcome HDL-C.


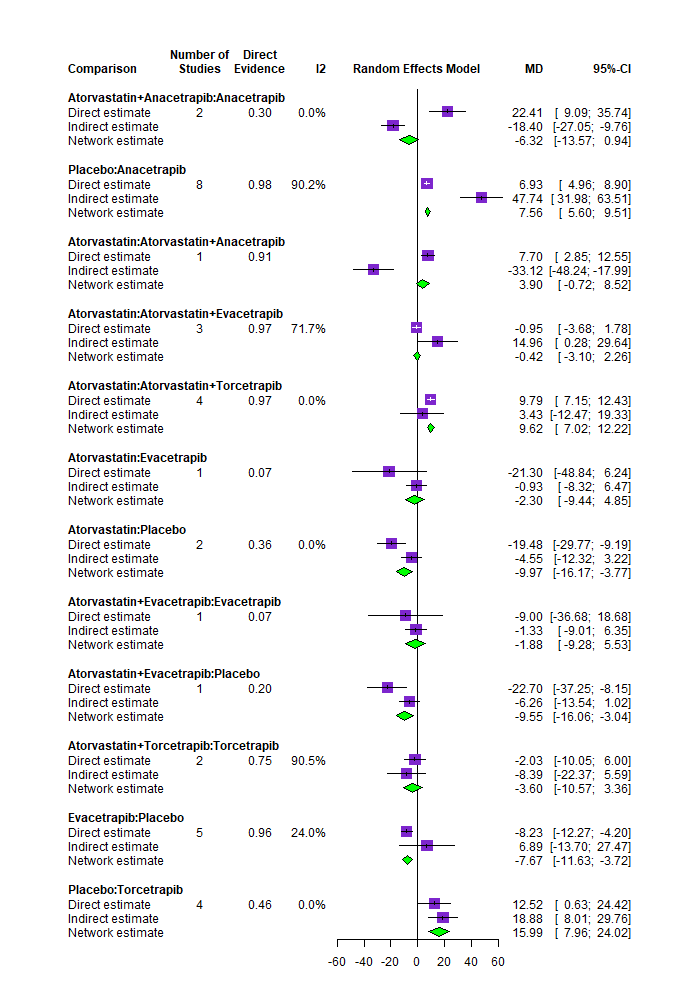


Supplementary Figure 9: Splitted forest plot showing differences among direct and indirect comparisons of intervention for the outcome TG.


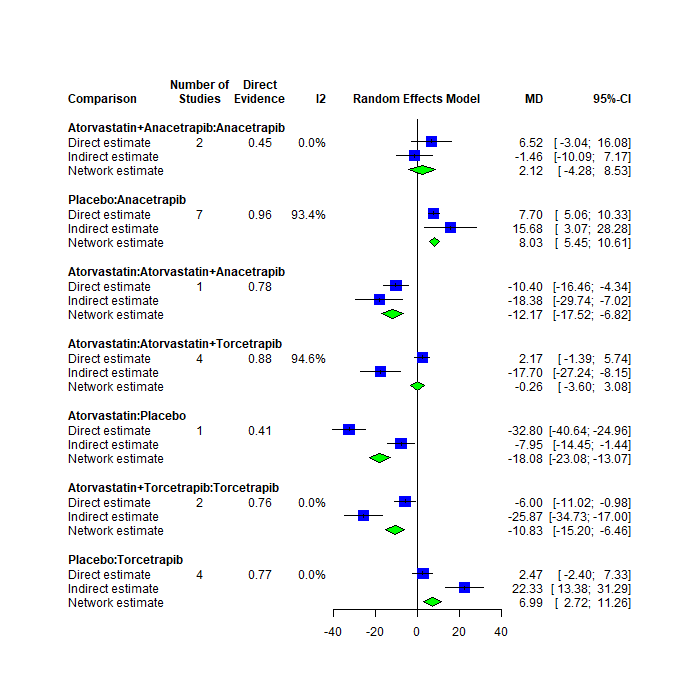


Supplementary Figure 10: Splitted forest plot showing differences among direct and indirect comparisons of intervention for the outcome TC.
